# Systematic review of *Coxiella burnetii* infection in humans, domestic and wild animals, ticks and ectoparasites in Algeria 1935–2025

**DOI:** 10.1016/j.onehlt.2026.101515

**Published:** 2026-07-12

**Authors:** Loïc Epelboin, Anissa Desmoulin, Olivier Duron, Moustapha Diop, Pierre-Edouard Fournier, Elodie Rousset, Nassima Achour

**Affiliations:** a Department of Infectious and Tropical Diseases, Cayenne University Hospital, Cayenne, French Guiana; b Clinical Investigation Center CIC INSERM 1424, UA 17 Santé des Populations en Amazonie, Cayenne University Hospital, Cayenne, French Guiana; c Department of Infectious and Tropical Diseases, Clinical Investigation Center CIC INSERM 1410, Sites Sud, CHU de La Réunion, Groupe Hospitalier Sud Réunion, Saint-Pierre, La Réunion, France; d MIVEGEC, University of Montpellier, Centre National de la Recherche Scientifique (CNRS), Institut de Recherche Pour le développement (IRD), Montpellier, France; e Department of Infectious and Tropical Diseases, Dakar Principal Hospital, Dakar, Senegal; f French Reference Center for Rickettsioses, Q Fever and Bartonelloses; RITMES - IHU Méditerranée Infection, 19-21 Boulevard Jean Moulin, 13005 Marseille, France; g French Agency for Food, Environmental and Occupational Health and Safety (ANSES), Sophia Antipolis Laboratory, Animal Q Fever Unit, Sophia Antipolis, France; h Algiers Faculty of Medicine, University of Health Sciences, Algiers, Algeria

**Keywords:** Q fever, *Coxiella burnetii*, Zoonoses, Camelids, Ticks, Lice, Algeria

## Abstract

**Background:**

Q fever, caused by *Coxiella burnetii*, is a globally distributed zoonosis that remains largely underdiagnosed in Algeria. Since its first description in 1935, fragmented data have accumulated across human, animal, and vector hosts without comprehensive synthesis. This systematic review summarizes all available information on the occurrence of *C. burnetii* in Algeria from 1935 to 2025, across human, animal and ectoparasite compartments, and critically discusses potential reservoirs, vectors and knowledge gaps.

**Methods:**

Following PRISMA 2020 guidelines, we searched PubMed, Scopus, ScienceDirect and the Algerian national thesis repository. Studies reporting original data on *C. burnetii* in humans, livestock, pets, wildlife, ticks or lice were included. Data were extracted on study period, location, host species, diagnostic methods, prevalence and molecular typing.

**Results:**

Sixty-one studies met the inclusion criteria. Human cases consisted mainly of sporadic acute infections, endocarditis and obstetric complications, often diagnosed abroad, reflecting limited local testing. In livestock, serological and molecular surveys showed infection in cattle, sheep, goats and camels across 30 among 58 wilayas, with apparent seroprevalence ranging from 8 to 27% in cattle, 15–75% in small ruminants and up to 70% in camels. Seventeen studies investigated arthropods: *C. burnetii* or *Coxiella*-like endosymbiont DNA was found in 13 tick species from ruminants, camelids and companion or wild animals, though the significance of these detections remains uncertain. Four studies reported on the bacterium in human and animal lice. Molecular typing identified several genotypes, suggesting multiple transmission cycles.

**Conclusions:**

Available evidence supports the long-standing and widespread circulation of *C. burnetii* in Algeria, but the true burden remains underestimated due to limited diagnostic capacity and lack of integrated surveillance. Strengthening local serological and molecular tools, including strain typing, is essential to clarify reservoirs, transmission dynamics and human infection sources within a One Health framework.

## Highlights

• First systematic review of Q fever in Algeria.
• Evidence of widespread underdiagnosis in humans and animals.
• Limited local diagnostic capacity hampers surveillance.
• High seroprevalence reported in livestock.
• Uncertain Tick molecular detections.
• Need for national One Health surveillance framework.

## 1 Introduction

Q fever is a zoonotic disease caused by *Coxiella burnetii*, a highly resistant intracellular bacterium with a global distribution [1]. It affects a wide range of hosts including ruminants, pets, wild animals and ticks, with humans acting as incidental hosts [2]. Transmission to humans primarily occurs through the inhalation of contaminated aerosols from infected animals, particularly during parturition [3]. In humans, the disease manifests in various presentations, ranging from pseudo-flu syndromes, community-acquired pneumonia to severe forms such as endocarditis and vascular infection. In animals, especially ruminant livestock, *C. burnetii* infection can cause abortion and stillbirth. For other reproductive disorders, such as infertility and reduced milk production, the evidence is limited to epidemiological associations rather than direct causation. Dedicated investigations are still needed to demonstrate causality. Abortion in livestock is a multifactorial condition that may result from a wide range of infectious causes—including *Brucella* spp., *Chlamydia abortus*, *Toxoplasma gondii*, *Salmonella* spp., *Listeria monocytogenes*, and *Campylobacter* spp.—as well as viral agents such as Rift Valley fever virus or Bluetongue virus, in addition to non-infectious factors (e.g., nutritional deficiencies, environmental stress, management practices); therefore, the detection of *C. burnetii* in abortion events should not be interpreted as evidence of causality without comprehensive differential diagnostic investigations. Nevertheless, Q fever is considered a potential contributor to significant economic losses in the livestock sector.

Ticks are also recognized as potential vectors of *C. burnetii*, and natural infections have been documented in several species [4], [5], [6]. However, interpretation of molecular detections is complicated by the widespread presence of *Coxiella*-like endosymbionts (CLE), which are genetically related but non-pathogenic [7], [8].

*C. burnetii* is recognized as a widespread but underrated worldwide pathogen including across the African continent. A systematic review on *C. burnetii* infection performed in 2014 highlighted substantial regional heterogeneity in seroprevalence among both animals and humans [9]. It should be noted that reported seroprevalence rates are based on studies with widely varying designs, sampling strategies, diagnostic methods, cut-off values, and population characteristics. As such, these figures are not directly comparable across areas, species, or time periods. For example, reported cattle seroprevalence varied from 4% to over 50%, while small ruminants frequently exhibited higher rates (11–33%). Human seroprevalence ranged from less than 8% in most regions to over 30% in specific subpopulations, such as children and rural dwellers in Egypt [10]. Thus, these figures reflect both true epidemiological differences and substantial methodological variability. Rather, they illustrate the overall heterogeneity and fragmentation of Q fever surveillance and research in Africa. This underlines the critical need for harmonized surveillance and standardized serological approaches.

In addition, Q fever was identified as the etiology of both non-specific acute febrile illness (2–9% of hospitalizations), and specific clinical presentations, such as 4 to 9% of community-acquired pneumonias (CAP) in some countries of Central Africa and in 1–3% of endocarditis cases across several African countries [11], [12], [13], [14]. However, these figures should not be interpreted as robust estimates of disease burden. The latter is probably underestimated due to limited access to the serodiagnosis and the molecular detection of *C. burnetii* in most countries of the continent, the limited incidence data and a lack of systematic diagnosis and surveillance efforts [15]. Despite the scarcity of data, *C. burnetii* is thought to be widely distributed across the African continent, both in wildlife and livestock, and little is known about the factors that promote or hinder bacterial circulation [1].

In Algeria, Q fever has long been considered underdiagnosed and underreported, with few diagnostic laboratories equipped to confirm infections [16]. This review aims to synthesize the historical and contemporary knowledge of C. burnetii infection in humans, livestock, companion animals, wildlife, ticks, and other ectoparasites in Algeria by integrating evidence from published human and veterinary studies, case reports, sero-epidemiological and molecular investigations, academic theses, and other publicly available grey literature spanning more than 90 years.

## 2 Methodology

### 2.1 Review of literature

A systematic review was conducted in accordance with the PRISMA 2020 guidelines [17]. We searched PubMed, ScienceDirect, and Scopus from inception to October 1st, 2025, using the MESH words (“Algeria” OR “Algiers”) AND (“*Coxiella burnetii*” OR “Q fever”), entering all keywords in both English and French to maximize retrieval. For grey literature, we systematically explored the national repository https://theses-algerie.com, which aggregates a large body of academic work—including unpublished dissertations and theses. Whenever both a peer-reviewed article and its underlying thesis manuscript were available, the published article was preferentially included, and the thesis was considered as duplicate, except if some interesting data had not been included in the publication. Records whose full text could not be accessed online—most often older, pre-digital publications—were excluded. We also excluded narrative or systematic reviews and knowledge–attitudes–practices surveys conducted among specific professions such as veterinarians or livestock breeders. However, a small number of narrative reviews were retained when they provided unique historical data whose primary publications were no longer retrievable.

### 2.2 Data analysis

Following study selection and analysis, results were grouped into four domains reflecting the One Health framework: (1) *C. burnetii* infection in humans; (2) infection in livestock; (3) infection in non-livestock animals (wildlife, pets, and other vertebrates); and (4) detection of *C. burnetii* in ticks and other ectoparasites. The livestock category was further stratified into small ruminants (sheep and goats), cattle, equines, and camelids. Prevalence was generally reported as both individual seroprevalence (percentage of seropositive animals) and herd-level seroprevalence (percentage of herds with at least one seropositive animal), which provides insight into historical and current exposure patterns, but does not directly reflect infection dynamics or risk of transmission and enable a detailed understanding of infection exposure within and between animal populations. Polymerase Chain Reaction (PCR) detects *C. burnetii* DNA and may provide evidence of current infection or shedding depending on the biological matrix and sampling context, whereas serology indicates past or present exposure. Finally, we included a dedicated section for studies that attempted strain typing of *C. burnetii*, most commonly using the multispacer sequence typing (MST) method, to explore molecular epidemiology whenever such data were available.

### 2.3 Meta-analysis

An exploratory descriptive quantitative synthesis was conducted using the *metafor* package in the R software environment (version 4.3.3) to visualize the distribution of reported individual seroprevalence and PCR positivity estimates. Heterogeneity between studies was assessed using the I^2^ statistic with a significance threshold of alpha = 0.05. Subgroup analyses (by species, sampling type and analytical method) were performed in case of high heterogeneity. The use of random-effects models does not remove the underlying biological and methodological heterogeneity and pooled values were therefore interpreted cautiously. Formal assessment of publication bias was not considered appropriate because of the high methodological and biological heterogeneity of the included studies, which limits the interpretability of funnel plots in this context. An exploratory funnel plot is provided as supplementary material, but it should not be interpreted as a formal assessment of publication bias because of the marked heterogeneity across studies.

### 2.4 Maps

Based on the reviewed literature, we also developed a geographical map summarizing whether each taxonomic group (humans, livestock, non-livestock animals, ticks/ectoparasites) had been studied in each of Algeria's 58 wilayas (provinces or governorates). This allowed us to identify both well-studied and under-investigated regions across the country. Maps were generated using QGIS 3.40 to illustrate the wilayas and the number of studies conducted in each wilaya between 1935 and 2025 in humans, livestock, non-livestock animals, and ticks and ectoparasites.

### 2.5 Diagnostic tools

The microbiological diagnosis of *human Q fever* currently relies primarily on serological assays, particularly indirect immunofluorescence assay (IFA), which remains the reference method. In animal studies, ELISA is more commonly used for serological surveys, while conventional or real-time PCR targeting multicopy elements such as IS1111 is used for molecular detection. However, a variety of diagnostic and epidemiological approaches have been used over time in both human and animal studies, including ELISA, complement fixation tests (CFT), microscopy techniques such as Stamp staining, PCR-based detection in ticks, and molecular typing methods (e.g., MST and sequencing). These methods are summarized and compared in Table 1.

**Table 1:** Microbiological methods for the detection of *Coxiella burnetii.*

Diagnostic method	Principle	Main limitations	Validation status by species
IFA (Indirect Immunofluorescence Assay)	Detection of phase I/II antibodies (reference method in humans)	− Subjective interpretation (reader-dependent) − Lack of standardized cut-offs in animals − Cross-reactions possible	- Reference standard in humans - Used in livestock but not standardized - Not validated in camels, horses, wildlife
ELISA	Detection of anti-*C. burnetii* antibodies (high-throughput)	− Cut-off variability − Species-, kit- and context- -dependent sensitivity/specificity − Detects exposure, not active infection or shedding	- Commercial ELISAs widely used in domestic ruminants - Diagnostic performance and optimal cut-offs vary by species, kit and epidemiological context - No species-specific validation for camels or horses
PCR (conventional / qPCR, e.g. IS1111 target)	Detection of bacterial DNA (blood, placenta, milk, vaginal swabs)	− Variable sensitivity depending on sample type − PCR inhibitors (milk, feces) − Intermittent shedding − Cannot distinguish viability	- Widely used in livestock - Performance depends on matrix - No harmonized protocols across studies
PCR in ticks	Detection of *C. burnetii* DNA in arthropods	− Major issue: confusion with Coxiella-like endosymbionts (CLE) − IS1111 cross-reactivity − Positive results may reflect blood meal contamination rather than infection	- Not reliable without advanced methods - Requires sequencing / multilocus confirmation
Complement fixation test (CFT)	Detection of antibodies (historical method)	− Low sensitivity - Obsolete compared to ELISA/IFA	− Historical use only (limited current relevance)
Stamp staining / microscopy	Direct visualization in placental material	− Low sensitivity − Non-specific	- Historical use - Not recommended for epidemiological studies
Genotyping (MST, sequencing)	Molecular characterization of strains	− Requires PCR-positive samples − - Limited availability	− Research use only (not diagnostic)

CLE, Coxiella-like endosymbionts; CFT, Complement Fixation Test; DNA, Deoxyribonucleic Acid; ELISA, Enzyme-Linked Immunosorbent Assay; IFA, Indirect Immunofluorescence Assay; IS1111, insertion sequence 1111; MST, Multispacer Sequence Typing; PCR, Polymerase Chain Reaction; qPCR, quantitative Polymerase Chain Reaction.

### 2.6 Ethical aspects

No specific ethical approval was required for this study, as we only analyzed previously published or publicly available literature. This review was not registered in PROSPERO; registration is not mandatory for this type of literature review.

## 3 Results

### 3.1 *Coxiella burnetii* infection in humans

**Fig. 1:**
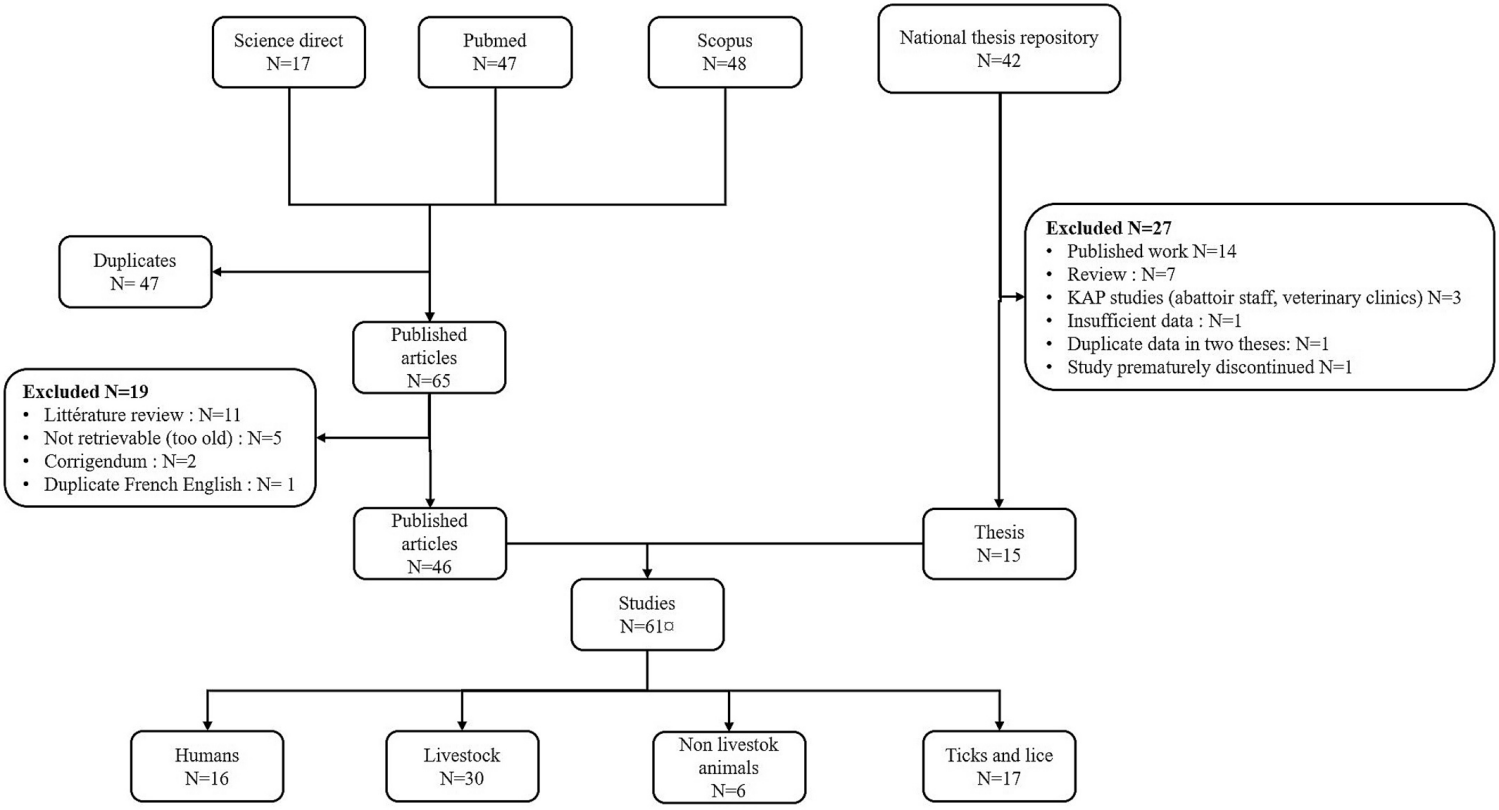
flowchart of the study. Systematic review of the literature following Prisma criteria. Because some studies include results on multiple host species, the total number of entries per group (humans, livestock, other animals, and ectoparasites) exceeds the actual number of individual publications.

Between 1935 and 2025, sixteen documents were used and analyzed for this review on human Q fever (Fig. 1).

#### 3.1.1 Historical cases and early observations

**Fig. 2:**
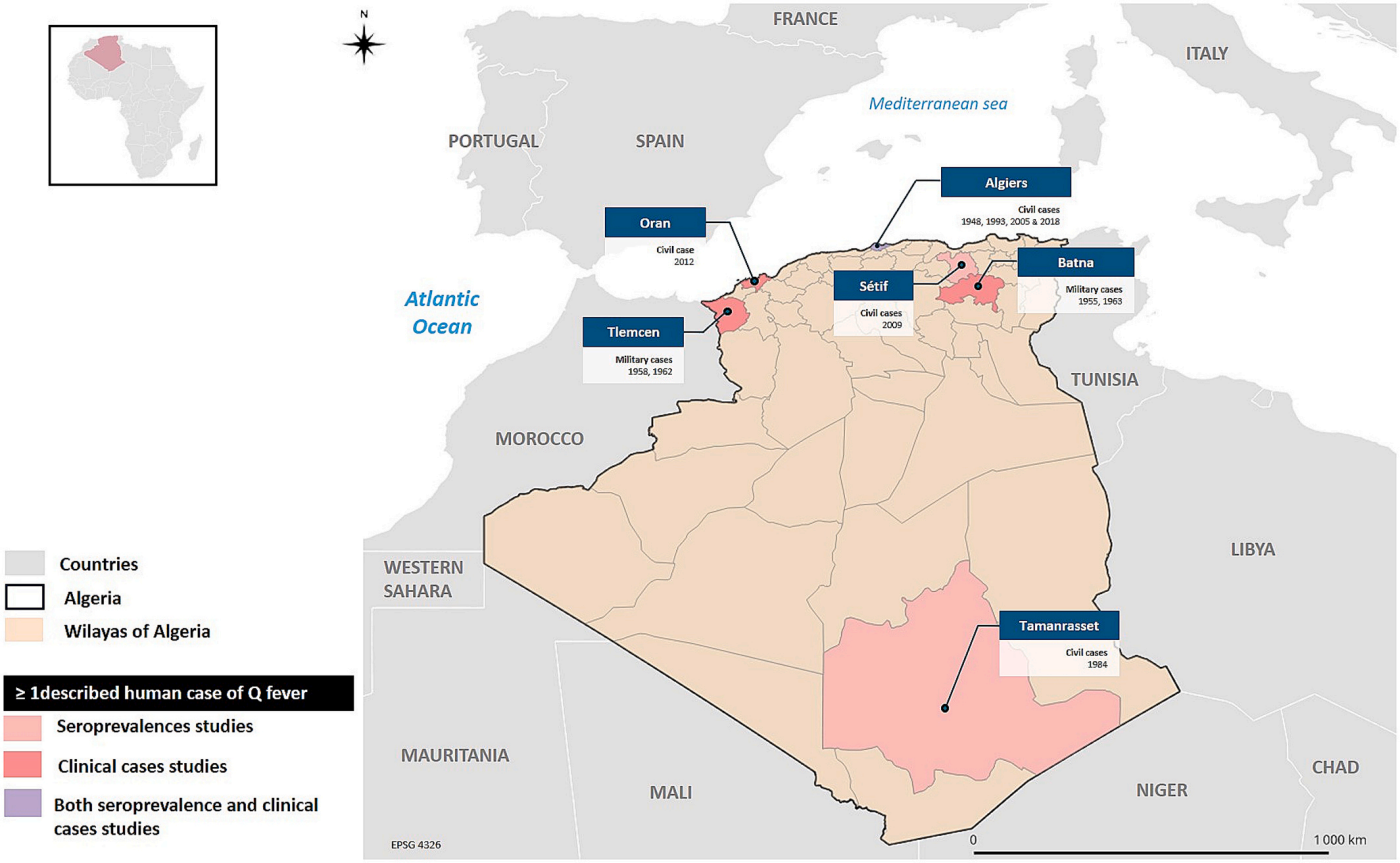
Map of Algeria representing the wilayas where studies on human Q fever were conducted between 1935 and 2025. Wilaya names are indicated together with the year of publication of the corresponding study and whether the investigated population was civilian or military. The map distinguishes seroprevalence studies (light pink), clinical case studies (dark pink), and wilayas where both types of studies have been reported (purple). (For interpretation of the references to colour in this figure legend, the reader is referred to the web version of this article.)

The earliest evidence of Q fever in humans in Algeria dates during the French colonization (1830–1962) (Table 2 and Fig. 2). The two first cases were documented in the region of Algiers in 1948 [18]. They presented as febrile syndromes with asthenia and nonspecific symptoms, consistent with what is now recognized as acute Q fever. Diagnosis at the time relied on clinical suspicion, and the laboratory diagnosis was possible for one of them. During the 1950s and early 1960s, several outbreaks involving an estimated 18 to 175 cases were documented among French soldiers in the Tlemcen and Batna regions, with transmission linked to livestock exposure [19], [20], [21], [22]. Bernard et al. (1963) discussed Q fever as a differential diagnosis in cases of unexplained febrile illness seen at Algerian hospitals, reflecting early awareness of its clinical relevance despite limited diagnostic tools [22].

**Table 2:** Synthesis of data in the medical literature on *Coxiella burnetii* infection in humans in Algeria.

1st Author	Year of the study	Wilaya / Region of Algeria	Biological methodology	Sample type	Type of population	Size of the sample	Result	Risk factors	Comments
Lengrand, 1948 [18] *	1948	Algiers	Serology	Blood	1 adult male patient 27 yo	1 + 1	Acute cases with fever, headache and CAP	None specified	Second human case of Q fever described in Algeria in 1948
Mimoune et al. 1955; Pierrou et al. 1955 [19], [20] *	1955	Batna	Serology	Blood	French militaries during the colonial era	175	Acute cases with fever, headache	Inhalation of dust in a van filled with manure and straw for transporting horses and brevis	Q fever fatigue syndrome
Lods et al. 1957 [21] *	1957	Batna	NA		French militaries during the colonial era	18	Acute cases	infiltration of animal manure from a neighboring barn into the dirt floor	
Bernard et al. 1963 [22]	1958	Tlemcen	NA		French militaries during the colonial era	22	Acute cases		
Bernard et al. 1963 [22]	1962	Tlemcen	NA		French militaries during the colonial era	24	Acute cases	sheep handling (meat and fleece)	
Dumas et al. 1984 [23] *	1978	Tamanrasset	Complement fixation	Blood	Nomadic and settled population (boys <16 years)	129	7 (5.4%) mais 7/54 (12.9%) mâle <16 ans	Contact with camelscamels, rural life	
Tien Nguyen et al. 1993 [25] *	1992	Algiers	Serology (IFA), histopathology	Blood, cardiac valve	Algerian woman in Paris	1	1	IE on Prosthetic valve, prior RHD	Patient supported in Paris
Auzary et al. 2001 [26] *	1990–1995	NA	Serology (IFA), histopathology, immunofluorescence on valves	Blood, cardiac valves	Algerian patients in Paris	6	5	IE on Prosthetic valve, prior RHD	Retrospective French hospital-based series
Benslimani et al. 2005 [28] *	2000–2003	Algiers	Serology (IFA) and Light Cycler nested-PCR	Serum	Patients with IE	108	2/108 (2%)	Not specified	Study focused on blood culture-negative IE in Algeria diagnosed in France
Lacheheb et al. 2009 [24] *	1995–1996	Sétif	Serology (IFA)	Blood	General population	729	113 (15.5%)	Being a rural inhabitant living in four highly endemic villages, professional exposure (not detailed)	First general population seroprevalence study in Algeria
Angelakis et al. 2014 [31] *	2012	Oran	IS1111 and IS30a RT PCR	Blood	Febrile patients	268	1 (0.3%)	21 yo man with an atypical CAP	Study among 1888 patients in 6 African countries
Ghaoui et al. 2018 [29] *	2014–2015	Algiers	IFA and IS1111 and IS30a RT qPCR	Serum, placenta	Women with febrile spontaneous abortion	725 women (380 cases, 345 women who gave birth without any other infections or complications)	3/380 positive in serology and 4/376 qPCR positive in cases; controls all negative	Animal contact, rural residence	Case-control study, link to obstetric complications
Louni et al. 2018 [33] *	2014–2016	Algiers, Tizi Ouzou, Boumerdès	IS1111 & IS30A qPCR, sequencing Cox 2, 5, 18, 22	Lice	Homeless persons	Total *n* = 44 Algiers *n* = 19 Tizi Ouzou *n* = 16 Boumerdes *n* = 9	2/44 (6%) 2/19 (10%) 0/16 (0%) 0/9 (0%)	Algiers vs Tizi Ouzou & Boumerdes	
Louni et al. 2018 [32] *	2016	Algiers	IS1111 & IS30A qPCR, sequencing Cox 2, 5, 18, 22		Nigerien refugees School children,	70 101	3 (4%) 0 (0%)		
Ghaoui et al. 2023 [30] *	2017	Several wilayas of Northern Algeria	IFA and IS1111 / IS30a qPCR	Blood	Patients with unexplained febrile illness	70 cases +70 controls without any ongoing infection	3/70 (4%) positive (2 with serology only and 1 with serology + PCR)	Contact with various animals including livestock	Acute Q fever in 3 with Prolonged unexplained fever including 2 with Meningeal syndrome
Ramos et al. 2024 [27] *	2023	ND	Serology (IgG phase II)	Blood, pericardial fluid	Algerian man in Portugal	1	Positive phase II IgG titer (1/512)	Farmer, contact with animals, ESRD	Case of Q fever pericarditis with tamponade in Algerian immigrant

Legend: * published study; ** non published thesis; CAP: community acquired pneumonia; RHD: rheumatic heart disease; IE: infective endocarditis; ESRD: End-Stage Renal Disease, ND: not detailed; IFA: Indirect Fluorescent Antibody test.

#### 3.1.2 Seroprevalence studies and population-based surveys

A turning point in epidemiological investigations came with the work of Dumas et al. in 1984, who conducted a wide serological survey in southern Algeria near Tamanrasset (Hoggar region) [23]. The study identified antibodies to *C. burnetii* among children under 16 years and various domestic animals, notably camels, sheep, and goats. It underscored the zoonotic risk in nomadic populations and confirmed the endemic nature of the bacterium in the region. The first structured seroprevalence survey in Algeria's general population was undertaken by Lacheheb et al. in 1995–1996 [24]. This cross-sectional study involved 729 participants in the wilaya of Sétif, a semi-arid agropastoral region of northeastern Algeria. Using immunofluorescence assay (IFA), the study reported a seroprevalence rate of 15.5%, with significant disparities between rural and urban settings. In four highly endemic villages, seroprevalence exceeded 30%. Risk factors associated with seropositivity included rural residence and occupational exposure, such as farming and animal husbandry. This pioneering work laid the groundwork for understanding Q fever epidemiology in Algeria. No further seroprevalence study in focused or general population has been performed since then. This gap highlights a major limitation in assessing the true seroprevalence of Q fever at the population level in Algeria.

#### 3.1.3 Endocarditis and chronic forms of Q fever

Q fever endocarditis has been documented in several Algerian patients supported in France and Portugal, often occurring in the context of valvular heart disease or prosthetic valves [25], [26], [27]. In addition, between 2000 and 2003, Benslimani et al. evaluated 108 Algerian patients of the region of Algiers with infective endocarditis and negative blood cultures, identifying two (2%) confirmed cases of Q fever endocarditis through serology and molecular tools performed in the French National Reference Center [28].

#### 3.1.4 Acute and obstetric presentations

In recent years, acute and obstetric forms of Q fever have been increasingly investigated in Algeria. Ghaoui et al. (2018) conducted a case-control study in two obstetric centers in Algiers, involving 725 pregnant women [29]. Among 380 women who experienced febrile spontaneous abortion, 4 tested positive by qPCR and 3 by IFA for *C. burnetii*. All controls were seronegative. While this study provided the first evidence of an association between Q fever and adverse pregnancy outcomes in Algeria, causality remains difficult to establish due to the limited number of confirmed cases and the absence of systematic investigation of alternative etiologies.

In another study, the same team (2023) examined 70 patients with unexplained febrile illness at the national infectious disease's hospital in Algiers [30]. Serology revealed three positive cases (4.3%), with one patient also testing positive by qPCR. These results suggest that Q fever may be an underappreciated cause of febrile illness of unknown origin in the Algerian hospital setting. Angelakis et al. (2014) identified one patient from Oran with a clinical picture of atypical community acquired pneumonia among 268 Algerian patients with unexplained fever in a multicountry African study on febrile illnesses [31]. At last, one study reported lice positive for *C. burnetii* found on homeless persons, Nigerien refugees and school children in Algiers in 2014 and 2016 [32], [33].

Strikingly, all confirmed human cases of Q fever in Algeria have been diagnosed abroad—either in migrants living in Europe or through retrospective testing of samples exported from Algeria. This dependence on foreign laboratories reveals a major gap in national diagnostic capacity, with direct consequences for both patient management and the reliability of epidemiological surveillance. As a result, Q fever is almost certainly underdiagnosed and underreported in Algeria.

Strengthening local diagnostic capacity therefore appears essential. Deploying serological and molecular tools at scale would allow timely diagnosis and treatment, including for severe or chronic forms such as endocarditis or pregnancy-related complications, while also supporting research into the country's true epidemiological burden.

In the short term, establishing partnerships to facilitate the referral and safe transport of clinical samples (UN2814) to national or international reference laboratories remains crucial for molecular confirmation, genotyping and strain characterization. Such measures will improve patient care and reinforce the surveillance system, paving the way toward sustainable national autonomy in Q fever diagnostics and research.

### 3.2 *Coxiella burnetii* infection in livestock in Algeria

As in most countries worldwide, the true burden of coxiellosis in livestock remains largely unknown in Algeria, owing to the absence of standardized etiological work-up procedures for reproductive disorders, as well as the lack of harmonized syndromic or event-based surveillance systems.

Over the past two decades, numerous studies have described the epidemiology of *C. burnetii* in Algerian livestock, mainly cattle, sheep, goats and camels, and to a lesser extent, horses. These investigations, using serological, molecular, and genotyping methods, provide a more detailed, yet still fragmented, picture of the distribution and risk factors of coxiellosis in animal populations across the country.

Overall, most studies are based on serological methods, whose inherent limitations (including the lack of a gold standard) preclude accurate estimation of true exposure rates. Ongoing work aims to optimise commercial Enzyme-Linked Immunosorbent Assay (ELISA) kit cut-offs for ruminant species [34], [35], but no species-specific validation is available for camels or horses, potentially resulting in over- or underestimation of seroprevalence in these animals. While serology indicates previous or current exposure, PCR-based studies provide evidence of active infection or shedding but are subject to variability owing to differences in limit of detection and matrix adaptation. The presence of PCR inhibitors in certain matrices, as well as the heterogeneous distribution of *C. burnetii* in tissues (e.g., placenta), may further affect both the comparability and the interpretation of detection rates. PCR results should also be interpreted according to the biological matrix: vaginal swabs, placental tissues, milk and blood do not provide equivalent information on infection, shedding or epidemiological risk.

Reported associations between seroprevalence and putative risk factors (e.g., odds ratios) should be interpreted as indicative trends rather than definitive evidence of transmission dynamics or disease risk factors. Because seroprevalence is a static marker of exposure rather than a direct measure of current infection or transmission, such associations serve primarily to guide further research, rather than to prove causality or establish dynamic risk. Such associations are useful for generating hypotheses and identifying contexts that deserve targeted investigation, but they should not be interpreted as proof of causal transmission pathways.

A total of 30 studies investigating *C. burnetii* infection in livestock were identified after elimination of duplicates (veterinary theses versus peer-reviewed publications) (Table 3). Of these, 13 focused on sheep, 12 on cattle, 7 on goats, 6 on camels, and 2 on horses. Q fever in livestock has been investigated in 30 of Algeria's 58 wilayas, with the highest number of reports from Médéa (*n* = 6), Béjaïa and Biskra (*n* = 5 each), Ain Defla and Ghardaïa (*n* = 4 each), Constantine, El Oued, Bordj Bou Arreridj, and Sétif (*n* = 3 each), Tiaret, El Bayadh, Blida, Ouargla, and Bouira (*n* = 2 each), and single studies reported from Tamanrasset, Sidi Bel Abbès, Jijel, Souk Ahras, Algiers, Tipaza, Boumerdès, Tébessa, Batna, Khenchela, M'sila, Laghouat, Mila, Guelma, El Tarf, and Tizi Ouzou (Fig. 3A, Fig. 3B).

**Table 3:** Synthesis of data in the medical literature on *Coxiella burnetii* infection in livestock in Algeria.

1st Author	Year of the study	Region of Algeria	Biological methodology	Sample type	Species of animal	Size of the sample	Result	Comments
Dumas, 1984 [23] *	1978	Tamanrasset	Complement fixation test (CFT)	Serum (filter paper elution)	Sheep, Goats, Camelids	Sheep *n* = 10 Goats *n* = 64 Camelids *n* = 25	Sheep 5/10 (50%) Goats 3/64 (5%) Camelid 6/25 (24%)	8 sheep came from Niger among which 3 were positive
Rahmani et al. 2013 [105] **	2013	Ain Defla & Medea	Stamp stain	Vaginal swab	Sheep, Goats (n = 6 in Ain Defla)	Sheep (*n* = 40; 15 in Ain Defla & 25 in Medea) Goats (n = 6 in Ain Defla & 0 in Medea)	Sheep 6 /40 (15%): 1/15 (7%) in Ain Defla & 5/25 (20%) in Medea Goats 2/6 (33%) in Ain Defla	Animals that have recently given birth or aborted
Rahmani et al. 2013 [105] **	2013	Ain Defla & Medea	Indirect ELISA	Blood	Sheep, Goats (n = 6 in Ain Defla)	Sheep (n = 40; 15 in Ain Defla & 25 in Medea) Goats (n = 6 in Ain Defla & 0 in Medea)	Sheep 8/40 (20%): 2/15 (13%) in Ain Defla & 6/25 (24%) in Medea Goats 1/6 (17%) in Ain Defla	Animals that have recently given birth or aborted
Yahyaoui et al. 2013 [46] **	2011	Medea	ELISA	Serum	Sheep	184 Sheep from 20 herds	8/20 (40%) positive herds 6/8 (75%) positive herds with abortions	No significant correlation between anti *C. burnetii* antibodies and abortion. No individual data available
Abdelkadir, 2020 [106] **	2013	Sidi Bel Abbes	Indirect ELISA	Serum	Sheep Herds with abortion disorders	180 Sheep from 39 herds	Individuals 50/180 (27.7%) Herds 28/39 (72%)	Higher infection rates in large herds than small herds
Hireche, 2020 [44] *	2011–2012	Constantine	Indirect ELISA	Blood	Sheep	226 from 39 herds	28 (12.4%) 14 (35.9%) herd seroprevalence	Risk factors: origin of animals, vaccination against enterotoxaemia and pox, access to the farm by foreign visitors, farmers/shepherds' visits to other farms, disinfection frequency and pest infestation within the farm.
Abdelhadi, 2015 [107] **	2013	Tiaret	ELISA	Serum	Cattle	92 from 40 herds	48/92 (41%) 22/92 (24%) positive 16/92 (17%) equivocal	
Khaled, 2016 [54] *	2011–2013	Medea, Djelfa, Ain Defla, Constantine, Bordj Bou Areridj, Biskra	Indirect ELISA, IS111 qPCR, MLVA (17 VNTR) Isolation on SFT cells	Serum	Sheep and Goats animals that have aborted or given birth within the last 8 days	227 sera, Among 29 herds	32/227 (14.1%) individuals 17/29(59%) herds 11.8% in Djelfa, 11.8% in Constantine, 19.2% in Biskra, 13.5% in Medea (52% herd) 13.9% of Sheeps 15% of Goatss	All 6 wilayas with positive cases. Risk factors of positivity in herds: history of abortion.
Khaled, 2016 [54] *	2011–2013	Medea, Djelfa, Ain Defla, Constantine, Bordj Bou Areridj, Biskra, Skikda, El Bayadh	IS111 qPCR, MLVA (17 VNTR) Isolation on SFT cells	vaginal swabs		267 swabs Among 35 herds	57/267 (21.3%) individuals 21/35 (61%) herds swabs + in PCR, 11/162 (6.8%) pos in abortions in Sheeps 0/35 (0%) pos in abortions in Goatss Ain Defla 7/7 (100%) 20.1% of Sheeps 30.2% of Goatss	11 novel MLVA genotypes (Table 5)
Sellali, 2016 [48] **	2015	Ain Defla, Medea, Djelfa, Biskra	Indirect ELISA	Serum	Sheep	102 sheep from 15 flocks 70 abortions 32 births	12/102 (11.8%) individual 7/15 (46.7%) flock-level 9/70 13% 3/32 9% Medea 3/47 (6%) Ain Defla 0/8 (0%) Djelfa 4/23 (17%) Biskra 5/24 (21%)	Primiparous more exposed; weak correlation with abortion
Radja, 2015 [43] **	2015	Jijel	Indirect ELISA	Serum	Cattle	184	29/184 (15.8%)	Altitude linked to *C. burnetii* infection; no significant link with abortion
Agag et al. 2016 [108] *	2015	Bejaia	ELISA	Serum	Cattle	180 cows from 50 herds	19/180 (10.6%) 11/50 (22%) herds positive	significant correlation with a history of placental retention, metritis, or regular return to estrus
Aouadi et al. 2017 [79] *	2014 & 2015	Souk Ahras	IS1111 & IS30a qPCR	Blood	Sheep, Goats	Sheep 120 Goats 120	7 (5.8%) Sheep 2 (1.7%) Goats	
Benaissa, et al. 2017 [61] *	2016	Biskra, Ouargla, Ghardaia, El Oued	Indirect ELISA	Serum	Camelids	184 camels from 31 herds	132 (71.2%) 26 (85.3%) herd-level seroprevalence Ouargla 28/43 (65.1%) Biskra 32/45 (71.1%) El-Oued 32/42 (76.2%) Ghardaia 40/54 (74.1%)	Risk factors: females, age > 11 years, herd size >50, tick infestation
Derdour et al. 2017 [39] *	2016	Algiers	ELISA, PCR	Serum	Cattle	360: 278 non-aborting and 82 aborting	6 (1.7%)	Significant higher number of positive in cow with abortion
Rahal et al. 2018 [42] *	2013–2015	Blida, Medea, Bouira & Bordj-Bou-Arreridj	IS1111 qPCR, MST genotyping	Placenta	Cattle	73	14/73 (19.1%) Blida 9/18 (50%) Medea 5/12 (41.7%) Bouira 0/7 (0%) BAA 0/6 (0%)	Reported association with abortion history in dairy cows MST20 genotype
Djellata et al. 2019 [38]	2014–2016	Blida, Algiers, Tipaza & Boumerdès	Indirect ELISA	Serum	Cattle	368 cows from 124 farms	31 (8.4%) 20 (16.1%) herd-level seroprevalence	Risk factors: fewer inspections, absence of artificial insemination, no rodent control
Bensoukhal et al. 2019 [63] **	ND	Ghardaïa, Biskra, El-Oued	Not detailed	Milk	Camelids	Not specified	Detection of antibodies in camel milk	
Bouakal et al. 2019 [50] **	2019	Tébessa	Indirect ELISA	Serum	Sheep	184 from 23 flocks	70 (30.0%) 22 (95.6%) herd seroprevalence	Risk factor: abortion
Mahdid et al. 2019 [55] **	2019	Medea	ELISA	Serum	Goats	103 animals from 14 herds	11 (10.7%) 8 (57.1%) herd-level prevalence	10 to 50% of infected herds. Risk factors: large herds and goats under 5 years of age. Weak association between the presence of antibodies and abortions (12.5%).
Ansel et al. 2020 [56] *	2017–2018	Tiaret, El-Bayadh, Ghardaia	ELISA	Serum	Horses	182	18 (9.9%) Tiaret 3/93 (3.2%) El Bayadh 13/70 18.6% Ghardaia 2/19 (10.5%)	Risk factor: Proximity of horses with camelscamels and small ruminants
Bellabidi et al. 2020 [60] *	2018–2019	Ouargla, El Oued, Biskra	Indirect ELISA and qPCR (IS1111 gene)	Blood	Camelids	184 camels from 12 herds	139 (75.5%) Biskra 90% Ouargla 84.6% Ouargla 65.7% 0/138 (0%) in qPCR	Risk factors: geographic location, age class, season
Menadi et al. 2020 [40] *	2016–2018	Setif	Indirect ELISA	Serum	Cattle	678 cows from 90 herds	77 (11.4%) 41 (45.6%) herd prevalence	Risk factors: contact with other herds, purchased animal; protective factor: use of disinfectants
Larab, 2020 [51] **	2020	Bejaia	Indirect Immunofluorescence	Serum	Sheep	15 males of slaughterhouse	6 (40%)	
Ansel, 2021 [58] **	2016–2019	Multiple wilayas (ND)	ELISA	Serum	Horses, Cattle, Sheep, Goats, Camelids	370 Horsess, 276 cattle, 550 sheep, 196 goats, 574 camels	Seroprevalence: 13.8% Horses 24.3% Cattle 43.5% Sheep 38.8% Goats, 57.3% camelids	Higher risk: small herds, female, tick presence, extensive farming
Guidoum, 2021 [109] **	2015–2016	Batna, Khenchela, Setif	Indirect ELISA	Blood	Cattle	344 cows from 22 herds	46 (13.3%) individual 11 (50%) herd prevalence	Risk factors: unprotected visitors, season, and water source were protective
Zemmouri et al. 2020 [45] *	2016–2018	M'sila	Indirect ELISA	Serum	Sheep	184 from 16 flocks	50 (27.2%) 16 (100%) herd seroprevalence	Risk factors: Stillbirth occurrence and cat presence
Bessas et al. 2022 [62] *	NA	Laghouat	IS1111 & *IS30A* qPCR	Blood	Camelids	80 from 3 herds	0 (0%)	Tested for several vector-borne pathogens, only other bacteria found
Menadi et al. 2022 [41] *	2017–2018	Setif	ELISA, IS1111a PCR, MST genotyping	BTM & blood	Cattle	200 BTM from 200 dairy herds, 186 blood from 26 herds	BTM: 74/200 (37%) ELISA+, 18/200 (9%) PCR+ Blood 13/186 (7.0%); 6/26 (23.1%) herds	Very low agreement ELISA/PCR (k = 0.08) MST12, MST32, new MST detected
Bento et al. 2023 [53] *	2020–2022	Mila, Constantine, Guelma, & El Tarf	Indirect ELISA	Serum	Goats	504 samples from 77 herds	44/504 (8.7%) 27/77 (35.1%) herd-level seroprevalence Mila 14/218 (6.4%), Constantine 23/142 (16.2%), Guelma 6/118 (5.1%), El Tarf 1/26 (3.9%)	Higher in herds with history of abortions (88.9%) Suburban environment > rural environment (16.1% vs. 7.3%)
Agag et al. 2023 [37] *	2022	Bejaia & Tizi Ouzou	Indirect ELISA	Bulk tank milk	Cattle	184 samples from 184 farms (Bejaia 92, Tizi Ouzou 92)	49/184 (26.6%) 10/184 doubtful 29/92 (31.5%) in Bejaia 20/92 (21.7%) in Tizi Ouzou	Risk factors: cohabitation with small ruminants, prevailing winds, veterinarian visit frequency
Ghaoui et al. 2024 [72] *	2017	Bejaia & Bouira	IS1111 & IS30A qPCR, MST genotyping	Blood, placenta, liver, spleen, uterus	Cattle, Sheep	Cattle: 120 blood, 75 liver, 75 spleen, 70 uterus Sheep: 84 blood, 35 placenta	Cattle Blood 3 (2.5%), liver 3 (4%), spleen 0 (0%), uterus 0 (0%) Sheep 1 (1.2%), placenta 3 (8.6%)	MST 33 (cattle, sheep), MST 20 (cattle)

Legend: * published study; ** non published thesis; BTM: bulk tank milk; BBA: Bordj Bou Arreridj; ND: not detailed; ELISA: Enzyme-Linked Immunosorbent Assay; MST: Multispacer Sequence Typing; IS1111 & IS30A: insertion sequences 1111 and 30 A; qPCR: quantitative Polymerase Chain Reaction.

**Fig. 3A:**
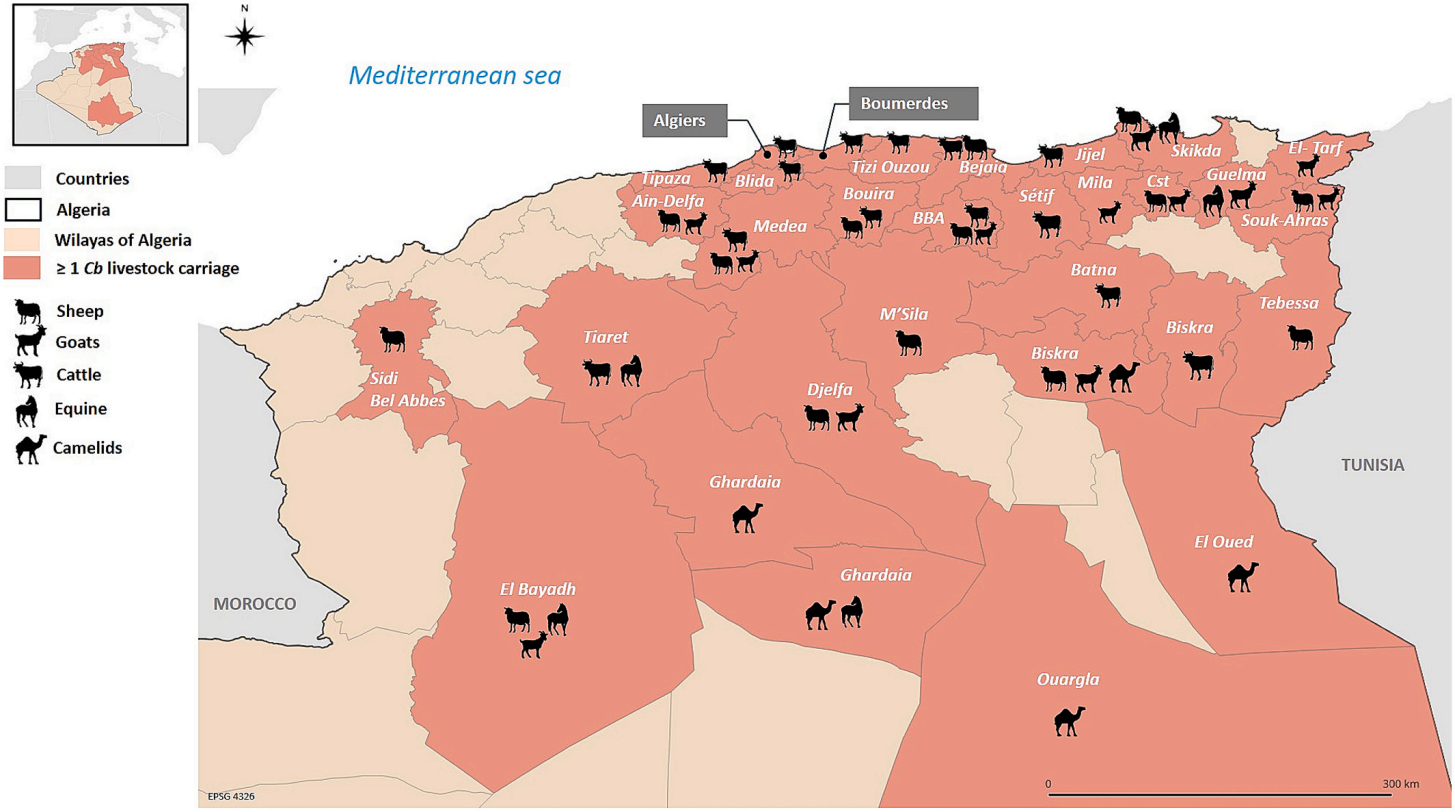
. Map of Northern Algeria representing the wilayas where studies on *Coxiella burnetii* infection in livestock were conducted between 1935 and 2025. Wilayas highlighted in dark orange indicate areas where at least one study was performed. Animal icons indicate the livestock species investigated in each wilaya (sheep, goats, equines, and camelids). BBA: Bordj Bou Arreridj; Cst: Constantine. (For interpretation of the references to colour in this figure legend, the reader is referred to the web version of this article.)

**Fig. 3B:**
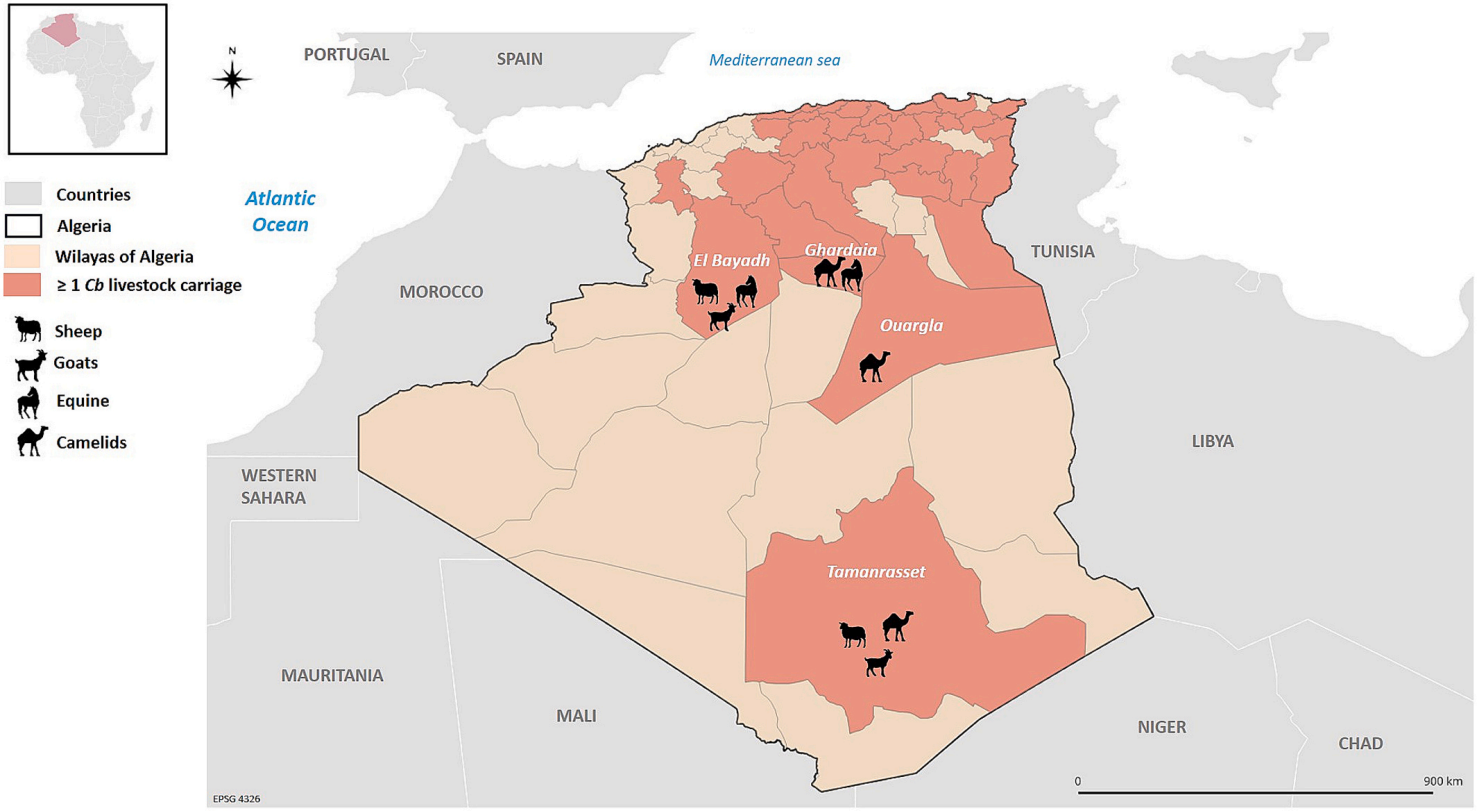
. Map of Algeria representing the wilayas where studies on *Coxiella burnetii* infection in livestock were conducted between 1935 and 2025. Wilayas highlighted in dark orange indicate areas where at least one study was performed. Animal icons indicate the livestock species investigated in each wilaya (sheep, goats, equines, and camelids). (For interpretation of the references to colour in this figure legend, the reader is referred to the web version of this article.)

Fig. 4A, Fig. 4B, Fig. 4C, Fig. 4D show the individual seroprevalence, the individual PCR positivity rate in blood, and the herd-level seroprevalence reported in the various identified studies, according to animal species.

**Fig. 4A:**
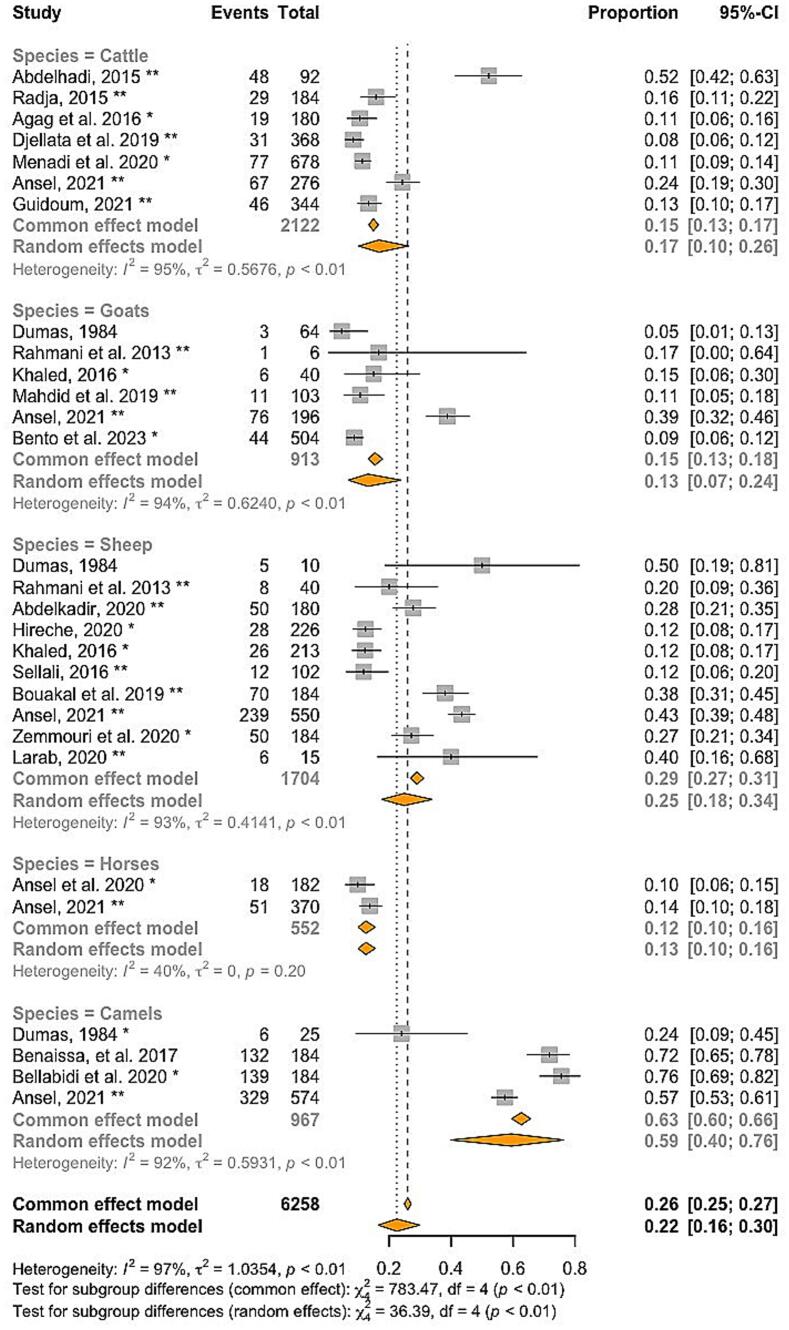
. Individual seroprevalence of *Coxiella burnetii* in livestock in Algeria according to the literature 1935–2025 according to the species (cattle, goats, sheep, horses and camels).

**Fig. 4B:**
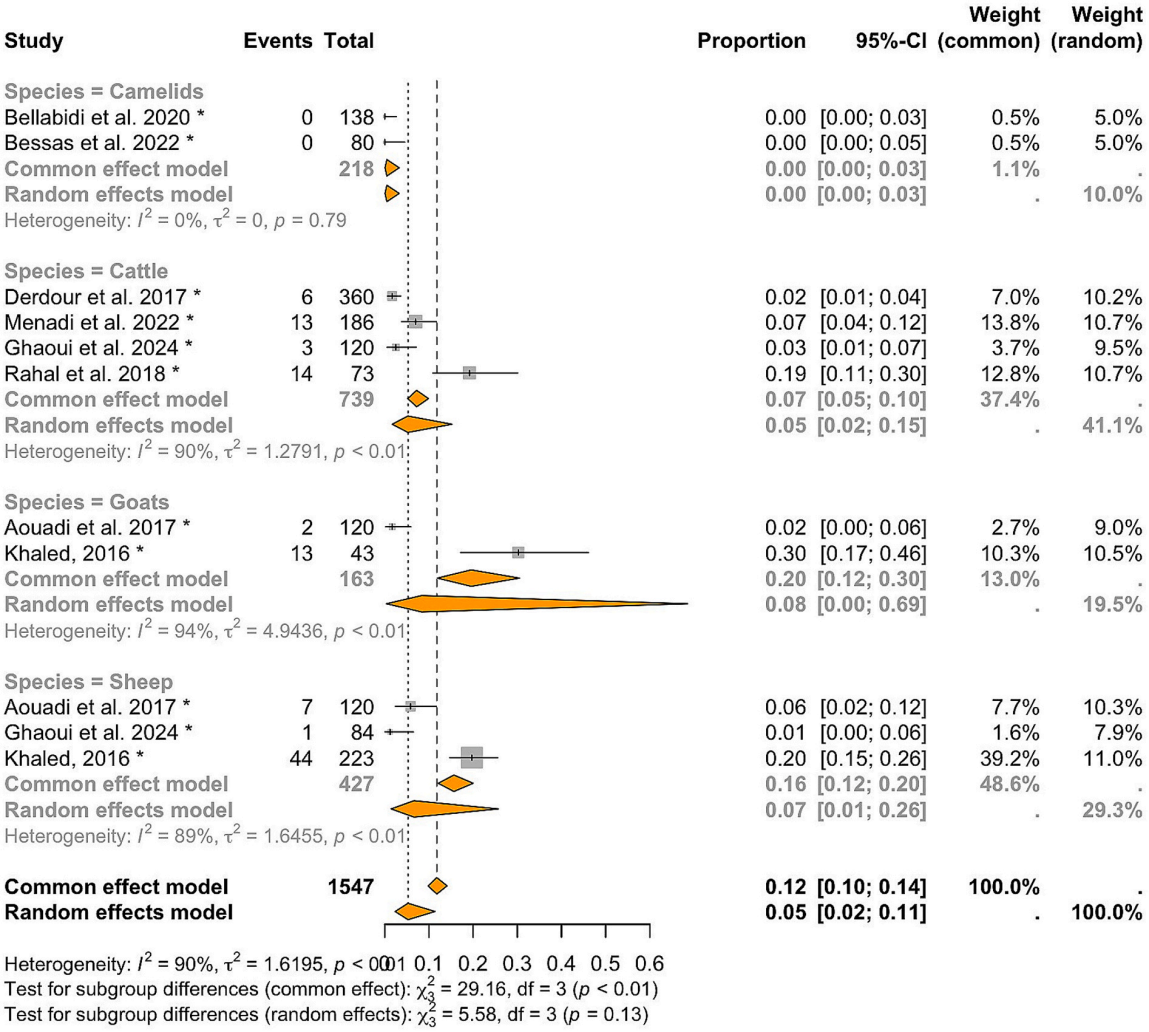
. Prevalence of *Coxiella burnetii* by Polymerase Chain Reaction in livestock in Algeria according to the literature 1935–2025 according to the species (cattle, goats, sheep, horses and camels).

**Fig. 4C:**
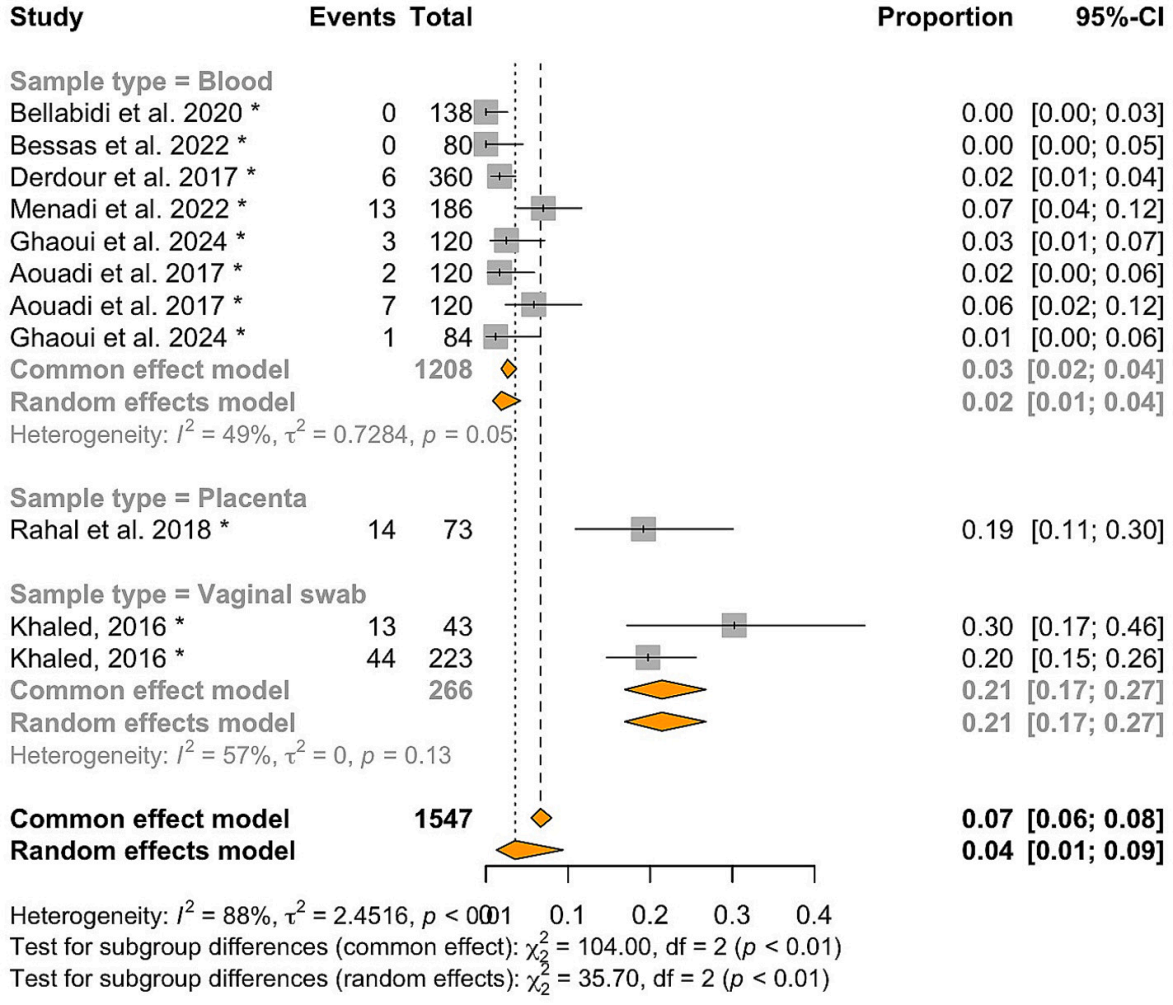
. Prevalence of *Coxiella burnetii* by Polymerase Chain Reaction in livestock in Algeria according to the literature 1935–2025 according to the sample (Blood, placenta and vaginal swabs).

**Fig. 4D:**
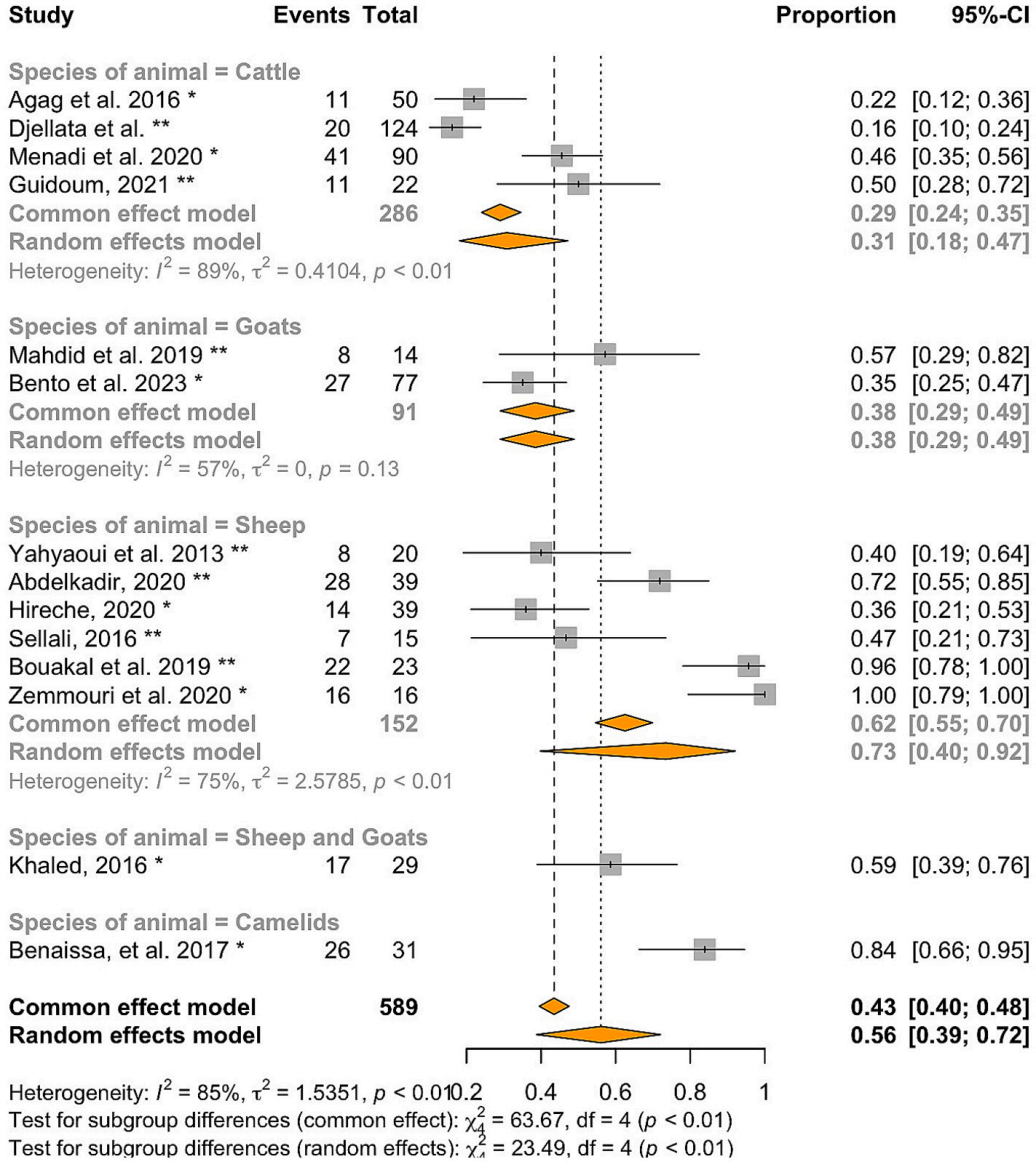
. Herd seroprevalence of *Coxiella burnetii* in livestock in Algeria by ELISA according to the literature 1935–2025 according to the species (cattle, goats, sheep and camels).

#### 3.2.1 *Coxiella burnetii* infection in cattle

In Algerian cattle, seroprevalence of *C. burnetii* has been found to vary widely across regions and herds. One of the most comprehensive studies was conducted by Agag et al. (2024), who reported a seroprevalence of 26.6% in bulk tank milk (BTM) samples collected from 184 dairy farms in the Kabylia region [36], [37]. The authors identified several risk factors, including cohabitation with small ruminants, prevailing winds, and insufficient biosecurity measures such as infrequent veterinary visits. Earlier, Djellata et al. (2019) investigated 368 cows from 124 farms in northern Algeria and found an individual seroprevalence of 8.4% and a herd-level prevalence of 16.1% [38]. Lack of artificial insemination and absence of rodent control were identified as significant risk factors. Similarly, Derdour et al. (2017) highlighted a significant association between *C. burnetii* seropositivity and bovine abortions in Algiers [39]. In the Setif region, Menadi et al. (2020) reported a seroprevalence of 11.4% among 678 cows from 90 herds [40]. A follow-up study by the same group (Menadi, 2022) using both ELISA and PCR revealed that 37% of BTM samples were ELISA-positive, while 9% were PCR-positive, with low concordance between the two techniques (kappa = 0.08) [41]. In Blida and Medea, Rahal et al. (2018) studied placental tissues from 77 cows and detected *C. burnetii* DNA in 19.1% of cases, mostly associated with abortion [42]. Finally, a study by Radja et al.(2015) in Jijel revealed a seroprevalence of 15.8% among 184 cows, and although no significant link was found between seropositivity and reproductive disorders, altitude appeared to influence infection risk [43].

#### 3.2.2 *Coxiella burnetii* infection in sheep

In sheep, *C. burnetii* appears to circulate extensively, with several studies documenting moderate to high levels of seroprevalence. Thus, Hireche et al. (2020) reported an individual seroprevalence of 12.4% and a herd-level prevalence of 35.9% in 226 ewes from Constantine [44]. Risk factors included visits from external personnel, lack of pest control, and incomplete vaccination protocols. A higher prevalence was found by Zemmouri et al. (2020) in M'Sila, where 27.9% of 184 ewes tested positive [45]. Interestingly, the presence of cats on farms was statistically associated with infection (OR = 5.75). In Ksar Boukhari, Yahyaoui et al. (2013) found that 75% of abortion-positive flocks were seropositive, although the correlation between infection and abortion was not statistically significant [46]. Lahrir et al. (2017) observed in an unpublished thesis particularly high levels of infection, with 75% seroprevalence among 184 ewes in southeastern Algeria, where abortion history and inter-herd contact were key predictors [47]. Similarly, Sellali et al. (2015) documented in another unpublished thesis a 12% seroprevalence in 102 sheep from four central wilayas, noting that primiparous ewes were most at risk [48]. In the Sidi Bel Abbes region, Abdelkadir et al. (2014) found 27.7% of ewes to be seropositive, while Bouakal et al. (2019) and Larab et al. (2020) also reported evidence of infection, including in small flocks and in rams [49], [50], [51]. Finally, Zemmouri et al. (2021) confirmed a 27.2% individual seroprevalence and noted mixed infections involving *C. burnetii*, *Chlamydia abortus*, and *Brucella* spp. [52]*.*

#### 3.2.3 *Coxiella burnetii* infection in goats

Although less frequently studied, goats have shown consistently high rates of infection. Bento et al. (2023) conducted a study in northeastern Algeria and reported an individual seroprevalence of 8.7% and a herd-level prevalence of 35% [53]. In herds with a history of abortion, this rate reached 88.9%. Khaled et al. (2016) reported a 14.1% seroprevalence in goats and found 21.3% of vaginal swabs to be PCR-positive [54]. At the herd level, 60% showed evidence of shedding. In Médéa, Mahdid et al. (2019) observed a seroprevalence of 10.7% among 103 goats from mixed-species herds, with younger animals being more frequently infected [55]. The same high level of seroprevalence (75%) observed in sheep by Lahrir et al. (2017) was also found in goats from the same region [47]. There is no published data on the seasonality of *C. burnetii* infection in livestock in Algeria.

#### 3.2.4 *Coxiella burnetii* infection in horses

Equines are not primary hosts for *C. burnetii*, but serological studies suggest occasional exposure. Ansel et al. (2020) reported the presence of antibodies in horses, though methodological details were limited [56]. The lack of species-specific validation for horses, may result in over- or under-estimation of true exposure rates [34], [35], [57]. In a more comprehensive study, Ansel (2021) reported a seroprevalence of 13.8% among 370 equines [58]. Risk factors included tick infestation, extensive grazing, and the nearby presence of other ruminants.

#### 3.2.5 *Coxiella burnetii* infection in camels

Dromedary camels, simply called camels, (*Camelus dromedarius*) have recently emerged as potential reservoirs of *C. burnetii* in the arid and Saharan regions of Algeria [59]. Several sero-epidemiological studies reported high seroprevalence rates, ranging from 57.3% to 75.5% depending on the region and study design [58], [60], [61]., although interpretation remains limited by the lack of validated serological assays and species-specific cut-offs for camels [34], [35]. PCR-positive ticks collected from camels further support active circulation of *C. burnetii* and suggest a vectorial role for *Hyalomma* and *Rhipicephalus* ticks. Risk factors associated with seropositivity included older age, large herd size, tick infestation, extensive or nomadic husbandry systems, and close contact with sheep and goats [58], [61].

In contrast, one study from Laghouat et al. did not detect *C. burnetii* DNA in camel blood samples by PCR [62], possibly reflecting regional differences or intermittent bacteremia. Detection of antibodies in raw camel milk [63] has raised concerns regarding foodborne transmission, especially given the widespread consumption of unpasteurized camel milk and fermented dairy products in southern Algeria. However, oral transmission of Q fever to humans remains unproven [64]. Traditional practices involving therapeutic use of camel urine may also represent a potential route of exposure [65].

Overall, these findings suggest that camels may contribute to the maintenance and environmental dissemination of *C. burnetii* through ticks and shedding in birth products, milk, feces, and urine. Given their major cultural and economic importance in Algeria, camels should be integrated into national Q fever surveillance and One Health strategies to better assess their epidemiological role and reduce zoonotic risk. Indeed, the current vaccine available for ruminants shows only moderate preventive efficacy: it is authorized in Europe for the three main ruminant species, demonstrating satisfactory protection in goats, uncertain effectiveness in cattle, and approval for sheep but with a limited duration of immunity of only several months [66], [67], [68].

### 3.3 *Coxiella burnetii* in non-livestock animals in Algeria

**Fig. 5:**
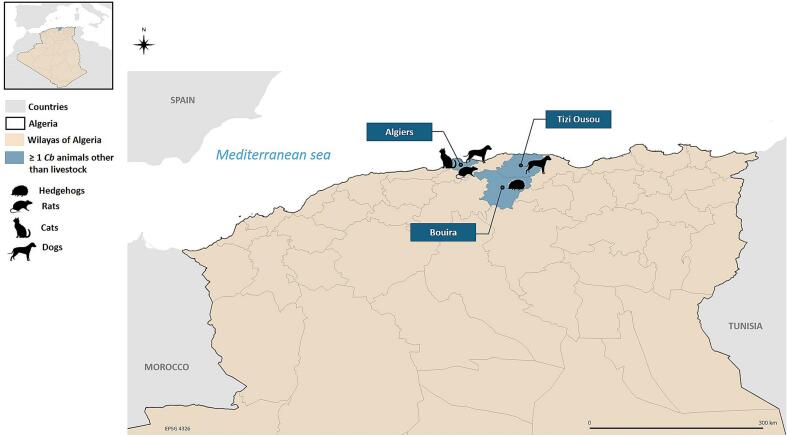
Map of Northern Algeria representing the wilayas where studies on *Coxiella burnetii* infection in non-livestock animals were conducted between 1935 and 2025. Wilayas highlighted in dark blue indicate areas where at least one study was performed. Animal icons indicate the species investigated in each wilaya (hedgehogs, rats, cats, and dogs). (For interpretation of the references to colour in this figure legend, the reader is referred to the web version of this article.)

While ruminants are recognized as the primary reservoirs for human *C. burnetii* infection, increasing evidence highlights the involvement of other mammals, domestic and wild species, in the ecology and epidemiology of *C. burnetii*. In Algeria, a few investigations have documented the presence of the pathogen in domestic carnivores, small wild mammals, and their ectoparasites (Fig. 5). Thus, 5 studies have been identified including dogs (*n* = 3), cats, bats, rats and hedgehogs (*n* = 1, each) (Table 4). These animal hosts may play an underestimated role in the maintenance and transmission of the bacterium, particularly in peri-urban and urban environments where human-animal interactions are common.

**Table 4:** Synthesis of data in the medical literature on *Coxiella burnetii* infection in animals except livestock in Algeria.

1st Author	Year of the study	Region of the study	Biological methodology	Sample type	Species of animal	Size of the sample	Result	Comments
Mailloux, 1962 [69], [70] *	1961	Algiers	Animal intraperitoneal inoculation, microscopy	Organs (brain, spleen)	Rat (*Rattus* sp.)	100	2/100 (2%) strains positive	
Bessas, et al. 2016 [71] *	2010–2013	Algiers	IS1111 qPCR	Spleen	Dog, Cat	117 dogs, 107 cats	1/117 (1.87%) dog and 1/107 cat positive for *C. burnetii*	
Aouadi et al. 2022 [74] *	2014–2017	Bouira	IS30A PCR	Kidney, spleen	Hedgehog (*Atelerix algirus*)	21 hedgehogs	6/20 spleen and 11/21 kidney positive	
Almandounas, 2023 [73] **	2022–2023	Tizi Ouzou	RT-PCR and IFI	Blood	Dog	6 dogs	0/6 (0%) positive in RT-PCR and 2/6 (33%) positive in IFI	
Ghaoui et al. 2024 [72] *	2017	Algiers	IS1111 and IS30A. qPCR and MST genotyping (Cox2, Cox5, Cox18, Cox20, Cox22, Cox37, Cox56, Cox57, and Cox61	Blood	Dog, Cat	80 dogs, 60 cats	10% dogs, 5% cats positive for *C. burnetii*	MST21 genotype identified in dogs and cats

Legend: * published study; ** non published thesis;

Historical, evidence for *C. burnetii* infection in wild rodents in Algeria was provided by Mailloux et al. (1962 & 1963) [69], [70]. In a survey of 100 rats (species not specified) captured in Algiers between April and July 1961, “*Rickettsia burneti”* (an earlier designation for *C. burnetii*) was isolated from two individuals through intraperitoneal inoculation into guinea pigs. The infected animals exhibited classical signs of coxiellosis including splenomegaly and typical intracellular rickettsial inclusions confirmed by Macchiavello staining. These early findings underscore the long-standing endemicity of *C. burnetii* in Algerian urban rodent populations.

A foundational study by Bessas et al. (2016) examined spleen samples from 117 stray dogs and 107 cats collected in Algiers between 2010 and 2013 [71]. Using qPCR, the authors detected *C. burnetii* DNA in one dog (0.85%) and one cat (0.93%). Both positive animals appeared clinically healthy but were parasitized by ticks and fleas. A more extensive survey was conducted by Ghaoui et al. (2024), who analyzed 599 biological samples (including blood, placenta, spleen, liver, and uterus) from cattle, sheep, dogs, and cats in northern Algeria [72]. Among 80 dogs and 60 cats sampled, qPCR revealed the presence of *C. burnetii* DNA in 10% of dogs and 5% of cats. This relatively low qPCR rate of 5% may be balanced by the supposed actual exponential and uncontrolled growth of the populations of stray cats in Algerian towns. Further confirmation of *C. burnetii* infection in dogs was provided by Almandounas and Bedrane (2023), whose Master's degree work focused on tick-borne pathogens in the region of Tizi Ouzou [73]. Ticks were collected from dogs housed in traditional shelters, and both the ticks and canine blood samples were tested using real-time PCR. Positive cases of *C. burnetii* were identified, underscoring the regional circulation of the pathogen among domestic carnivores and their ectoparasites. A detailed investigation by Aouadi et al. (2022) targeted the North African hedgehog (*Atelerix algirus*) in the Bouira region, north-central Algeria [74]. Over a three-year period (2014–2017), 21 hedgehog carcasses and their ectoparasites (ticks and fleas) were collected and analyzed using molecular methods. *C. burnetii* DNA was detected in hedgehog spleens and kidneys, as well as in their associated ectoparasites, including *Rhipicephalus sanguineus* sensu lato, *Haemaphysalis erinacei*, and the flea *Archaeopsylla erinacei*. This highlights the potential for hedgehogs to act as amplifying hosts and for their parasites to serve as vectors for zoonotic transmission. Bats have also been implicated in the ecology of *C. burnetii* in Algeria. Leulmi et al. (2016) identified the bacterium in 3 out of 19 *Ixodes vespertilionis* ticks collected from bats (*Chiroptera* spp.), in the northeastern provinces of El Tarf and Souk Ahras [75]. This corresponds to a positivity rate of 15.8%. Although *C. burnetii* was not detected directly in bat tissues in this study, the presence of the pathogen in their hematophagous ectoparasites supports the possibility of a sylvatic transmission cycle involving bats and ticks. However, the detection of *C. burnetii* in ticks is technically challenging and should be interpreted with caution, as discussed in the following section [8], [76].

### 3.4 Detection of *Coxiella burnetii* in ticks and lice in Algeria

#### 3.4.1 Ticks

Several investigations have explored the presence of *Coxiella burnetii* in ticks and ectoparasites in Algeria (Figs. 6A & B). Overall, 17 studies investigated *C. burnetii* in at least 13 tick species, including *Argas persicus*, *Dermacentor* spp., *Haemaphysalis erinacei*, *Hyalomma* spp., *Ixodes vespertilionis*, *Rhipicephalus bursa*, and *R. sanguineus*, as well as human body and head lice (Table 5). These ectoparasites were collected from cattle, sheep, goats, camels, poultry, dogs, cats, bats, hedgehogs, and other wild and domestic animals.

**Fig. 6A:**
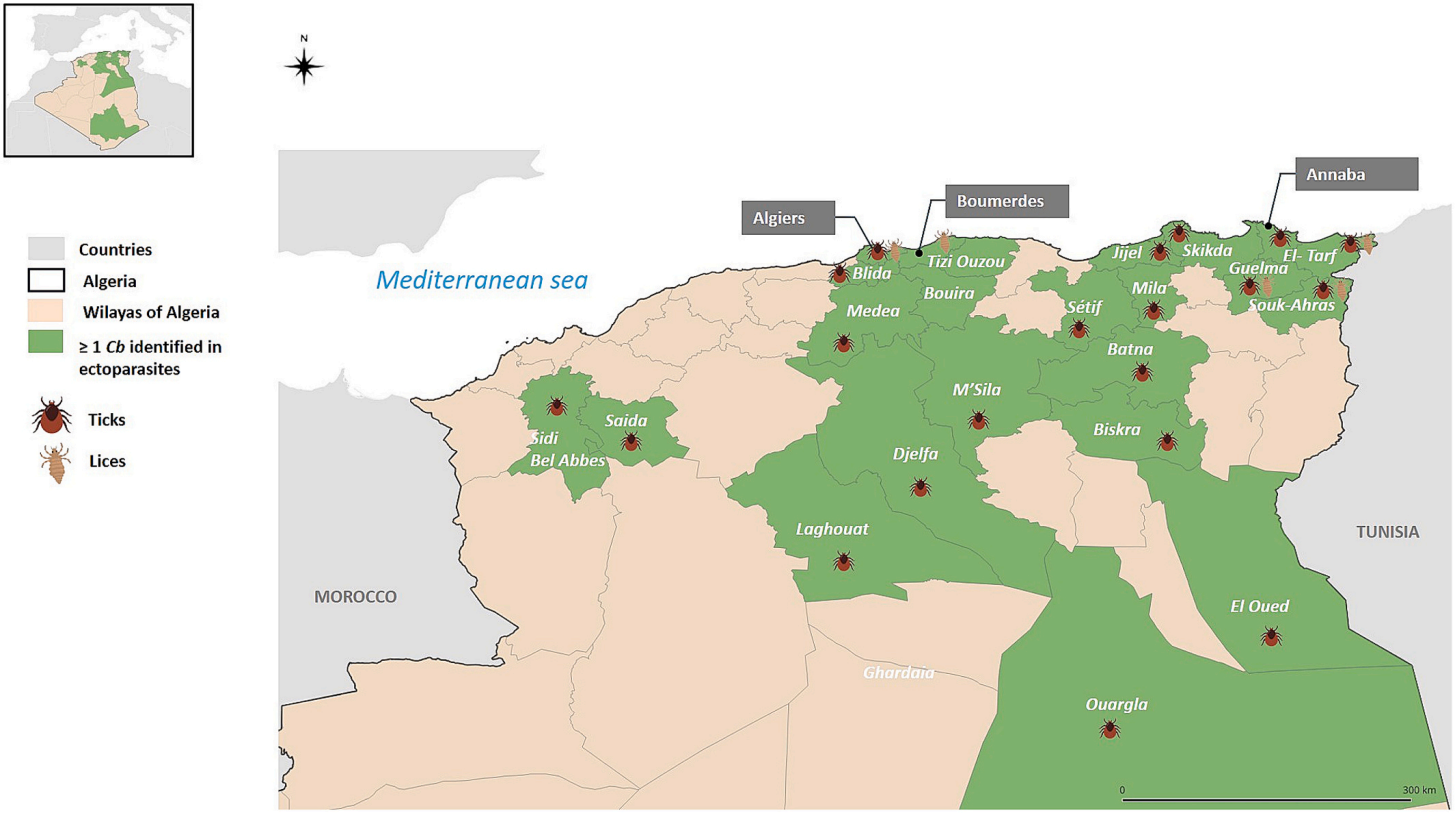
Map of Northern Algeria representing the wilayas where studies on *Coxiella burnetii* in ticks and lice were conducted between 1935 and 2025. Wilayas highlighted in green indicate areas where at least one study was performed. Ectoparasite icons indicate whether studies involved ticks and/or lice. (For interpretation of the references to colour in this figure legend, the reader is referred to the web version of this article.)

**Fig. 6B:**
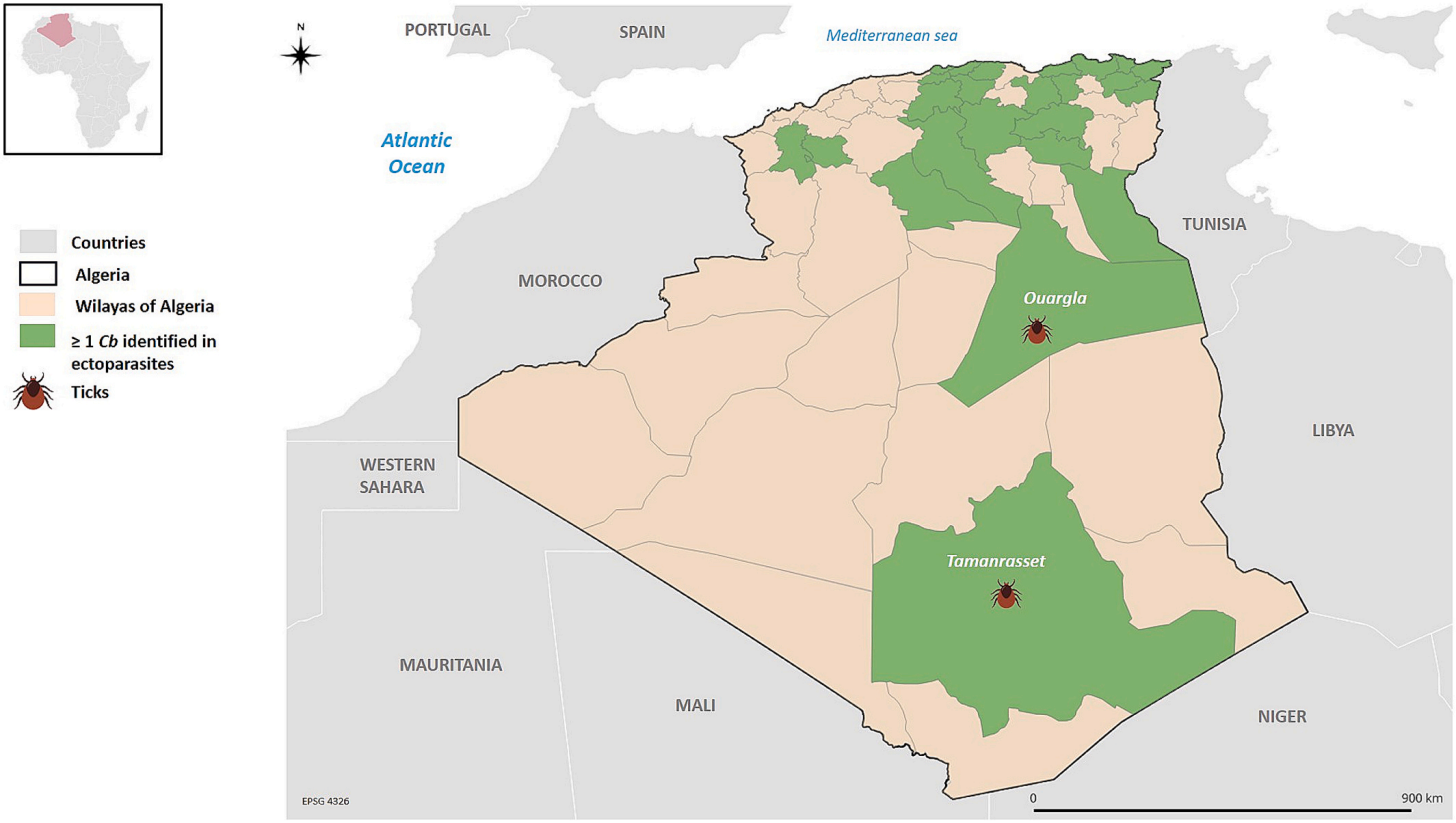
Map of Algeria representing the wilayas where studies on *Coxiella burnetii* in ticks and lice were conducted between 1935 and 2025. Wilayas highlighted in green indicate areas where at least one study was performed. Ectoparasite icons indicate whether studies involved ticks and/or lice. (For interpretation of the references to colour in this figure legend, the reader is referred to the web version of this article.)

**Table 5:** Synthesis of data in the medical literature on *Coxiella burnetii* infection in ticks and ectoparasites in Algeria.

1st Author	Year of the study	Region / Wilaya	Biological methodology	Sample type	Species	Size of the sample	Result	Comments
Blanc et al. 1949 [77] *	1948	Algiers	Inoculation of ticks in Gp and then injection of Gp spleen samples in other Gp. Smear and serodiagnosis	Ticks from cattle	*Hyalomma mauritanicum*	17 female	Smear-positive shred	
Bessas et al. 2016 [71]*	2010–2013	Algiers	IS30a and IS1111 qPCR	Ticks from stray dogs and cats	*Rhipicephalus sanguineus*	532	0/532 (0%)	
Leulmi, et al. 2016 [75] *	2012 & 2013	El Tarf & Souk Ahras	IS1111 & IS30a qPCR	Ticks from wild and domestic animals	*Ixodes vespertilionis* of bats *(Chiroptera* spp.)	191 ticks in El tarf 77 Souk Ahras	0 El Tarf 3 Souk Ahras 3/19 (16%) *I. vespertilionis*	High risk of false positives due to confusion with *Coxiella*-like endosymbionts, resulting from the lack of specificity in molecular assays
Aouadi et al. 2017 [79] *	2014 & 2015	Souk Ahras	IS1111 & IS30a qPCR	Ticks from sheep	*Rhipicephalus bursa*	73	4/73 (5%)	High risk of false positives due to confusion with *Coxiella*-like endosymbionts, resulting from the lack of specificity in molecular assays
Boucheikhchoukh et al. 2018 [110] *	2015–2016	9 wilayas (El Tarf, Annaba, Guelma, Souk Ahras, Skikda, Mila, Sétif, M'Sila & Jijel)	qPCR	Ticks from domestic and wild animals	9 tick species *(*e.g. *Rh. sanguineus, Hy. scupense)*	219	0 (0%)	
Louni et al. 2018 [33] *	2014–2016	Algiers, Tizi Ouzou, Boumerdès	IS1111 & IS30A qPCR, sequencing Cox 2, 5, 18, 22	Body lice of homeless persons	*Pediculus humanuscorporis*	Total *n* = 524 Algiers *n* = 235 Tizi Ouzou *n* = 184 Boumerdes *n* = 115	10/524 (1.9%) 10/235 (4.2%) 0/184 (0%) 0/115 (0%)	Algiers vs Tizi Ouzou & Boumerdes
Louni et al. 2018 [32] *	2016	Algiers	IS1111 & IS30A qPCR, sequencing Cox 2, 5, 18, 22	Head lice of Nigerien refugees and School children	*Pediculus humanuscapitis*- clade E lice	28/50 women refugees with lice 3/20 child refugees with lice ➔ 37 lice 27/101 school children ➔ 45 lice	3/37 (8%) 0/45 (0%)	
Abdelkadir et al. 2019 [111] *	2016–2017	Sidi Bel Abbes & Saida	16S rRNA PCR, specific PCR	Ticks from livestock (ruminants)	*Rhipicephalus bursa, Hyalomma excavatum, Hyalomma scupense & Hyalomma marginatum*	149 *R. bursa* (*n* = 74), *H. excavatum* (*n* = 68), *H. scupense* (n = 3) & *H. marginatum* (n = 4)	2 pools of *H. excavatum (Cattle/Sidi Bel Abbis;* Sheep/Saida) 2 pools of *R. bursa (Sheep/ Sidi Bel Abbis)*	High risk of false positives due to confusion with *Coxiella*-like endosymbionts, resulting from the lack of specificity in molecular assays
Bellabidi et al. 2020 [60] *	2018–2019	Ouargla, El Oued & Biskra	IS1111 qPCR, sequencing of the PCR std. products	Ticks from camels	*Hyalomma dromedarii,* *H. impeltatum,* *H. excavatum*	60 ticks 50 *Hyalomma dromedarii,* *9H. impeltatum,* *1H. excavatum*	7/60 (12%) 1/1*H. excavatum* 5/50*H. dromedarii*; *1/9H. impeltatum*	thresholds 24 ≤ Ct ≤ 35. Strains related to those in Tunisia, Greece, Mediterranean. High risk of false positives due to confusion with *Coxiella*-like endosymbionts, resulting from the lack of specificity in molecular assays
Ouchene et al. 2020 [83] *	2014–2015	Medea	IS30A qPCR	Ticks from chicken	*Argas persicus*	34	0 (0%)	
Rahal et al. 2020 [78]*	2013–2015	Blida, Medea, Batna,	qPCR, sequencing	Ticks from cattle	13 species including *Rhipicephalus ssp., Hyalomma ssp, Dermacentor ssp*	147	*Coxiella*-like endosymbionts in 51.7% (76/147); not *C. burnetii*	
Ouarti et al. 2021 [88] *	2015–2017	El Tarf, Souk Ahras, Guelma.	IS1111 & IS30A qPCR	Lice from 11 Cattle, 9 sheep, 5 goats and 6 poultry	*Linognathus vituli* (cattle) *Linognathus* *Africanus* (goat)	300 300	1 (0.3%) 3 (1%)	
Hamlili et al. 2022 [112] *	2018	Tamanrasset	*MALDI-TOF MS, IS30A and IS1111 qPCR*	Ticks from camels	*Hyalomma* spp./ *Hyalomma dromedarii* *H. excavatum* *H. impeltatum* *H. anatolicum*	91 ticks (41 males & 50 engorged females) from 9 camels 41 males 42 4 3 1	2/91 (2.2%) 1*H. impeltatum* & *H. dromedarii*	High risk of false positives due to confusion with *Coxiella*-like endosymbionts, resulting from the lack of specificity in molecular assays
Aouadi et al. 2022 [74] *	2020–2021	Bouira	qPCR	Lice and ticks from hedgehogs	*Archaeopsylla erinacei, Haemaphysalis erinacei, Rhipicephalus sanguineus s.l.*	229 ticks: 119 *A. erinacei*; 78 *R. sanguineus*; 32*H. erinacei*	4/119 *A. erinacei*; 9/78 *R. sanguineus*; 15/32*H. erinacei*	High risk of false positives due to confusion with *Coxiella*-like endosymbionts, resulting from the lack of specificity in molecular assays
Mana, 2022 [87] **	2020–2022	Northern Algeria (5 wilayas)	qPCR, standard PCR, sequencing	Lice from goats	*Linognathus africanus*	20	2/20 (10%)	
Almandounas, 2023 [73] **	2022–2023	Tizi-Ouzou	qPCR	Ticks from cattle, goats and dogs	*Rhipicephalus turanicus*, *R. bursa, R. sanguineus*	28 shred of ticks	1 *Rhipicephalus* *Sanguineus*	High risk of false positives due to confusion with *Coxiella*-like endosymbionts, resulting from the lack of specificity in molecular assays
Kautman, 2025 [82]*	2023–2024	Laghouat, Djelfa, El Tarf	qPCR	Ticks from 240 Greek tortoises (*Testudo graeca*)	*Hyalomma aegyptium*	Total *n* = 134 Laghouat *n* = 90 Djelfa *n* = 20 El Tarf *n* = 24	0 /134 (0%)	

Legend: * published study; ** non published thesis; Gp: Guinea pigs; qPCR (IS1111, IS30A): quantitative polymerase chain reaction targeting insertion sequences IS1111 and IS30A;

Several studies reported *Coxiella* DNA in ticks collected from cattle, sheep, and goats, particularly in *Hyalomma*, *Rhipicephalus*, and *Dermacentor* species from northern and highland pastoral regions [77], [78], [79]. Mixed sheep-goat flocks often harbored overlapping tick communities, suggesting that small ruminants may jointly contribute to the maintenance of *C. burnetii* in agro-pastoral ecosystems. Similar findings were reported in camel ticks from southern Algeria, especially *H. dromedarii*, *H. impeltatum*, and *H. excavatum*, with approximately 12% PCR positivity reported in one study [60]. Phylogenetic analyses suggested links with strains circulating in neighboring countries. *Coxiella* DNA was also occasionally detected in ticks collected from dogs, cats, bats, hedgehogs, and tortoises, suggesting the possible existence of a sylvatic cycle [73], [75], [80], [81], [82]. In contrast, soft ticks from poultry (*Argas persicus*) showed negative or very low detection rates [83].

However, these molecular findings require cautious interpretation (Table 6). A major limitation is the widespread presence of CLE, which are genetically closely related to *C. burnetii* but non-pathogenic [7], [8]. CLE are highly prevalent in ticks worldwide and may coexist with *C. burnetii*, making standard PCR assays insufficiently specific. In particular, IS1111-targeted PCR assays, widely used for Q fever diagnosis in vertebrates, may cross-react with sequences from some CLE genotypes and therefore artificially overestimate *C. burnetii* prevalence in ticks [84], [85]. More specific approaches, including validated qPCR targets, multilocus sequencing, or whole-genome analyses, are needed to accurately discriminate *C. burnetii* from CLE [8], [76], [86].

**Table 6:** Key limitations of PCR-based detection of *Coxiella burnetii* in ticks.

Confusion with Coxiella-like endosymbionts (CLE)	Many tick species naturally harbor *Coxiella*-like endosymbionts, which are genetically closely related to *C. burnetii*. Common PCR targets (e.g., IS1111) may amplify both organisms, leading to potential misidentification
Lack of specificity of commonly used targets	Single-locus PCR assays lack discriminatory power. Without confirmatory methods (e.g., sequencing, multilocus typing), it is often not possible to distinguish *C. burnetii* from related endosymbionts.
Detection of bacterial DNA does not imply infection or vector competence	PCR positivity does not demonstrate that ticks are infected, nor that they are capable of transmitting the pathogen.
Influence of blood meals (false interpretation)	Many studies analyze engorged ticks. Detection of *C. burnetii* DNA may simply reflect the presence of bacteria in the host blood meal rather than true colonization of tick tissues.
Absence of standardized methodologies	Differences in sampling strategies, DNA extraction protocols, PCR targets, and interpretation criteria limit comparability between studies.
Need for advanced molecular approaches	Reliable identification requires more specific methods such as multilocus sequence typing, target-specific qPCR assays, or whole-genome sequencing.

Interpretation of PCR results for *Coxiella burnetii* in ticks is subject to several major limitations that may lead to overestimation of its prevalence and epidemiological role.

Another important limitation concerns the frequent analysis of engorged ticks in Algerian studies. Detection of *C. burnetii* DNA in blood-fed ticks may merely reflect transient contamination from infected host blood rather than true infection of tick tissues or vector competence [8]. Consequently, current data do not allow firm conclusions regarding the role of ticks in Q fever transmission in Algeria. Experimental studies using unfed ticks and tissue localization approaches are required to better assess the epidemiological significance of ticks in the maintenance and transmission of *C. burnetii*.

Although numerous tick species have been found PCR-positive for *C. burnetii* worldwide, robust evidence supporting their role as competent vectors remains limited. Experimental and historical studies have demonstrated that some tick species, particularly within the genera *Dermacentor*, *Hyalomma*, *Rhipicephalus* and *Ornithodoros*, can maintain and transmit *C. burnetii* under laboratory conditions [5]. For example, *Dermacentor andersoni* and *Dermacentor variabilis* have been shown to transmit the bacterium transstadially and transovarially in experimental settings. Similarly, species such as *Hyalomma dromedarii* and *Rhipicephalus sanguineus* have been implicated in the maintenance of *C. burnetii* in enzootic cycles, although direct evidence of transmission to humans remains scarce. Despite these findings, the epidemiological role of ticks in natural transmission cycles is still considered secondary compared to aerosol transmission from infected animals [8]. Most field detections rely on PCR-based methods and are subject to the methodological limitations discussed above, particularly the inability to distinguish *C. burnetii* from CLE. Therefore, while some tick species may contribute to enzootic cycles under specific conditions.

#### 3.4.2 Lice

Several investigations have addressed the presence of *C. burnetii* in lice collected from humans and animals in Algeria (Fig. 6A, Fig. 6B). Early molecular work on body lice (*Pediculus humanus corporis)* from homeless individuals in Algiers, Tizi Ouzou, and Boumerdès detected *C. burnetii* DNA in 10 of 524 specimens (1.9%), infesting two of 44 (4.5%) participants, with positive lice belonging to haplotype A5 [33]. Subsequent surveys on head and body lice from Nigerien refugees and schoolchildren revealed *C. burnetii* sequences in up to 8% of head lice (*Pediculus humanus capitis)*, suggesting that imported clades may act as carriers [32]. In farm settings, *Linognathus africanus* from goats harbored *C. burnetii* in around 10% of specimens, while lice from cattle were mainly negative [87]. Additional sampling from cattle, sheep, goats, and poultry confirmed *Coxiella* DNA in some animal lice pools, marking the first evidence of Q fever agents in animal lice in Algeria [88]. Collectively, these findings indicate that both human lice and sucking lice of livestock (*Linognathus* spp.) can harbor *C. burnetii* or closely related organisms, although their epidemiological significance remains unclear. However, lice do not naturally harbor CLE, so the detection of *C. burnetii* in these vectors is less ambiguous than in ticks. Yet, because surveyed lice were engorged (as they were collected directly from hosts), the presence of *C. burnetii* DNA may reflect the ingested blood rather than an established infection and therefore does not necessarily indicate that lice are competent vectors. Further work is needed to determine whether lice merely reflect host bacteremia or might occasionally support the maintenance or transmission of *C. burnetii*.

### 3.5 Genotyping data of *C. burnetii* in Algeria

To date, several studies have reported molecular characterization of *C. burnetii* strains circulating in Algeria, using multispacer sequence typing (MST) (Table 7). These investigations revealed the presence of diverse genotypes in both domestic animals and companion species. Rahal et al. (2018) were the first to report the detection of MST20 in placental tissues from aborting cattle in the Blida and Médéa regions, highlighting a potential link with reproductive disorders [42]. A broader study by Ghaoui et al. (2024) identified three MST genotypes circulating in northern Algeria: MST20 and MST33 in cattle and sheep, and MST21 in domestic dogs and cats, underlining the potential role of companion animals in the epidemiological cycle [72]. Moreover, Menadi et al. (2022) described MST12, MST32, and a novel MST genotype in BTM and blood samples from dairy herds in northeastern Algeria, suggesting further genotypic diversity in the national livestock population [41]. Collectively, these findings indicate the co-circulation of multiple *C. burnetii* genotypes across regions and host species, but the clinical and epidemiological significance of this diversity remains to be established. To clarify the potential links between specific genotypes, animal reservoirs, and clinical outcomes—including reproductive disorders—future work should prioritize the systematic collection and molecular typing of isolates from both human and animal cases. The development of national or regional databases integrating molecular and epidemiological data will be essential to monitor trends, track outbreaks, and support One Health surveillance strategies.

**Table 7:** Genotyping data available in the literature on *Coxiella burnetii* in Algeria.

Reference	Host species	Sample type	Detection method	Identified MST	Region / wilaya
Khaled 2015 [113] **	Sheep & Goats	Vaginal swabs	MCLA	11 novel genotypes in MLVA: Dz-B7, Dz-M2, Dz-S1, Dz-M1, Dz-B1, Dz-B2, Dz-B2, Dz-B3, Dz-B4, Dz-B5, Dz-B6, Dz-BY1	Ain Defla, Biskra, Bordj Bouareridj, Constantine, Djelfa, El Bayadh, Medea, and Skikda
Rahal et al. 2018 [42] *	Cattle (placenta)	Placental tissue (aborted cows and calving cows)	qPCR (IS1111, IS30A) + MST (7 spacers)	MST20	Blida and Medea
Menadi et al. 2022 [41] *	Cattle	BTM, blood	PCR (IS1111) + MST (Cox2, 5, 18, 20, 22, 37, 51, 56, 57 and 61)	MST12, MST32, novel MST	Setif
Ghaoui et al. 2024 [72] *	Cattle	Blood, liver	qPCR (IS1111, IS30A) + MST (Cox2,5,18,20,22, 37,56, 57, 61)	MST20, MST33	Algiers, Bouira and Bejaia
	Sheep	Blood, placenta		MST33	Algiers, Bouira and Bejaia
	Dogs and Cats	Blood		MST21	Algiers

Legend: * published study; ** non published thesis; (MCLA: Microimmunofluorescence or Complement fixation assay for serological diagnosis; qPCR (IS1111, IS30A): quantitative polymerase chain reaction targeting insertion sequences IS1111 and IS30A; MST: Multispacer Sequence Typing.

## 4 Discussion

### 4.1 Regional perspective: North Africa and Sahelian context

The epidemiology of Q fever in neighboring North African countries suggests that *C. burnetii* is widely circulating across North Africa, although available data remain heterogeneous and fragmentary. In Morocco, human seroprevalence studies reported variable results ranging from 1% among blood donors in Casablanca to 18.3% in Fez, indicating substantial regional differences in exposure [89]. However, recent molecular investigations failed to detect *C. burnetii* DNA among febrile patients, highlighting the discrepancy frequently observed between serological and PCR-based studies in Africa [31]. In Tunisia, several studies documented particularly high levels of exposure in both humans and animals. Human seroprevalence reached 26% among blood donors in central Tunisia [90]. Animal studies demonstrated intense circulation of *C. burnetii*, especially in dromedary camels, with seroprevalence ranging from 44% to 73.6% [91], [92]. In addition, *C. burnetii* DNA was detected in *Hyalomma dromedarii* and *H. impeltatum* ticks collected from camels, supporting a possible role of ticks in transmission dynamics [93]. Sheep in Tunisia also showed significant seropositivity, further emphasizing the importance of livestock reservoirs in the region [94]. By contrast, no specific epidemiological study on Q fever could be identified from Libya, representing a major gap in the understanding of Q fever epidemiology in North Africa [9]. This absence of data likely reflects underdiagnosis, limited surveillance systems, and insufficient research rather than a true absence of circulation.

Overall, the epidemiological patterns observed in Morocco and Tunisia are consistent with findings from Algeria, particularly regarding the high seroprevalence observed in camels and the probable importance of extensive pastoral systems and tick exposure in maintaining *C. burnetii* circulation. These regional similarities support the hypothesis that Q fever is largely underrecognized throughout North Africa and highlight the need for coordinated One Health surveillance approaches integrating humans, livestock, wildlife, and vectors across this Northern area of the African continent.

In Sahelian countries, particularly those with extensive camel breeding such as Niger, Chad, and Mauritania, high seroprevalence rates have also been reported in dromedaries, supporting the hypothesis that camels may represent important reservoirs in arid and semi-arid ecosystems [95], [96], [97], [98]. As in Algeria, these findings must be interpreted with caution due to the lack of species-specific validation of serological tools and the heterogeneity of study designs.

Overall, the epidemiology of Q fever across North Africa and the Sahel appears to share common features, including high exposure in livestock, underdiagnosis in humans, and significant methodological heterogeneity. These similarities reinforce the need for coordinated regional approaches, including harmonized diagnostic tools, shared surveillance frameworks, and collaborative research initiatives to better understand the circulation of *C. burnetii* in these interconnected ecosystems.

### 4.2 Recent findings

Recent studies increasingly suggest a potentially important role of camels as hosts of *C. burnetii* in North Africa, the Middle East, and other arid regions. High seroprevalence rates have been reported in Algeria (57–76%), Tunisia (44–74%), Egypt (41%), Jordan (50%), Kenya (21–52%), and several Sahelian countries, supporting widespread exposure of camel populations to *C. burnetii* [60], [92], [95], [96], [97], [98], [99], [100], [101]. Molecular studies have also detected *C. burnetii* DNA in camel blood, vaginal swabs, abortion products, milk, and associated ticks, particularly *Hyalomma* spp. [60], [102], [103]. Risk factors consistently associated with infection include older age, tick infestation, extensive pastoral systems, herd mixing with small ruminants, and abortion history [92], [99], [100], [104]. Although these findings suggest an important role for camels in the ecology of Q fever, their interpretation remains limited by the lack of species-specific validation of serological assays and the heterogeneity of study designs. Together, these observations reinforce the need to integrate camel populations into One Health surveillance and research programs on Q fever [101], [104].

### 4.3 Limitations

#### 4.3.1 Lab methodology

A critical distinction must be made between exposure, infection and shedding when interpreting the available data. Serological assays detect antibodies and therefore reflect past or present exposure, but do not provide direct evidence of active infection or bacterial shedding. PCR detects bacterial DNA and may indicate ongoing infection or shedding depending on the sample analyzed; however, DNA detection does not necessarily imply viability or infectivity and may be influenced by sampling conditions and matrix-specific limitations. Therefore, most available data from Algeria should be interpreted primarily as indicators of exposure or molecular detection rather than direct measures of transmission risk.

#### 4.3.2 Geographical coverage and spatial limitations

The geographical distribution of studies on *C. burnetii* in Algeria reveals marked spatial heterogeneity, with several wilayas remaining entirely uninvestigated (“white areas”). These gaps likely reflect disparities in research capacity, accessibility, and local diagnostic infrastructure rather than the true absence of infection. As such, the absence of data in these regions should not be interpreted as absence of disease, but rather as a major limitation in current surveillance systems.

Beyond these structural gaps, environmental and ecological factors are likely to play a key role in shaping the distribution of Q fever in Algeria. Livestock density, particularly in regions with intensive sheep and goat farming, may favor the maintenance and amplification of *C. burnetii*. Pastoral and transhumance practices, which involve seasonal animal movements and inter-herd mixing, may further facilitate pathogen circulation across regions. In addition, climatic and environmental conditions—such as aridity, temperature, and especially wind patterns—are critical determinants of airborne dissemination, as *C. burnetii* is known to be highly resistant in the environment and can be transported over long distances through contaminated aerosols.

The available data also suggests the possibility of regional clustering of infection risk, particularly in northern and steppe regions where livestock production is more intensive and better studied. However, this apparent clustering must be interpreted with caution, as it may partly reflect a concentration of studies in these areas rather than true epidemiological hotspots. Conversely, southern and sparsely populated regions, including Saharan areas, remain under-investigated despite environmental conditions that could support pathogen persistence and transmission. Importantly, a substantial proportion of livestock data originates from veterinary theses conducted in proximity to the main veterinary schools located in Algiers, Constantine, Batna, Tiaret, Blida, and Souk Ahras. This academic structuring likely contributes to a spatial clustering of studies around these centers, further reinforcing geographic bias in the available data.

Altogether, these findings highlight the need for more geographically balanced surveillance strategies, integrating environmental, veterinary, and human health data, in order to better identify high-risk areas and understand the spatial dynamics of Q fever within a One Health framework.

## 5 Conclusion

This review highlights the need to move beyond fragmented data toward a structured and integrated national approach to Q fever surveillance in Algeria. A One Health framework—linking human, animal, and environmental health sectors—should be prioritized to better capture the complexity of *C. burnetii* transmission. Such a system would rely on coordinated surveillance of human cases (particularly febrile illness, endocarditis, and obstetric complications), systematic investigation of livestock reproductive disorders, and targeted monitoring of high-risk environments such as pastoral and peri-domestic settings.

Operationally, strengthening diagnostic capacity is a critical first step. This includes the wider deployment of validated serological assays and molecular tools at regional levels, the standardization of diagnostic protocols across laboratories, and improved access to confirmatory testing. The establishment of national or regional biobanks, integrating human, animal, and environmental samples, would support both surveillance and research efforts. In parallel, the development of molecular platforms, including whole genome sequencing (WGS), would enable strain characterization, source attribution, and better understanding of transmission pathways.

Capacity-building is equally essential. Training programs for clinicians, veterinarians, microbiologists, and public health professionals should be implemented to improve awareness, diagnosis, and reporting of Q fever. Strengthening collaboration between academic institutions, reference laboratories, and public health authorities will be key to ensuring sustainability.

Based on the current evidence, several public health priorities emerge. First, improving the diagnosis and recognition of human Q fever, particularly in cases of unexplained febrile illness, endocarditis, and adverse pregnancy outcomes. Second, implementing standardized surveillance in livestock, especially in small ruminants and camels, which appear to play a major role in pathogen circulation. Third, addressing major knowledge gaps through coordinated epidemiological and environmental studies, particularly in under-investigated regions.

Ultimately, the implementation of a coordinated, multidisciplinary, and geographically balanced surveillance system will be essential to accurately assess the burden of Q fever in Algeria and to design effective prevention and control strategies within a One Health framework.

## Author contributions (CRediT taxonomy)

Conceptualization: LE.

Methodology: LE, MD.

Data curation: LE.

Formal analysis: MD.

Investigation: LE.

Visualization: AD.

Writing – original draft: LE.

Writing – review & editing: LE, AD, MD, OD, PEF, ER, NA.

Supervision: OD, PEF, ER, NA.

Project administration: LE.

Resources: LE

## CRediT authorship contribution statement

**Loïc Epelboin:** Writing – original draft, Validation, Supervision, Resources, Methodology, Investigation, Formal analysis, Data curation, Conceptualization. **Anissa Desmoulin:** Writing – review & editing, Visualization. **Olivier Duron:** Writing – review & editing, Validation. **Moustapha Diop:** Methodology, Formal analysis. **Pierre-Edouard Fournier:** Writing – review & editing, Validation. **Elodie Rousset:** Writing – review & editing, Validation, Methodology. **Nassima Achour:** Writing – review & editing, Validation, Supervision.

## Declaration of competing interest

The authors declare that they have no known competing financial interests or personal relationships that could have appeared to influence the work reported in this paper.

## Data Availability

Data will be made available on request.

## Appendix A Supplementary data

Supplementary Figure S1. Funnel plot assessing potential publication bias among the studies included in the meta-analysis.
